# Probiotics for the Treatment of Bacterial Vaginosis: A Meta-Analysis

**DOI:** 10.3390/ijerph16203859

**Published:** 2019-10-12

**Authors:** Ziyue Wang, Yining He, Yingjie Zheng

**Affiliations:** 1 Department of Epidemiology, School of Public Health, Fudan University, Shanghai 200032, China; 2 Department of Health Policy and Management, School of Public Health, Peking University Health Science Center, Beijing 100191, China; 3 China Center for Health Development Studies, Peking University, Beijing 100191, China; 4 Key Laboratory of Health Technology Assessment, National Health Commission (Fudan University), Shanghai 200032, China; 5 Key Laboratory of Public Health Safety, Ministry of Education, School of Public Health, Fudan University, Shanghai 200032, China

**Keywords:** probiotic therapy, bacterial vaginosis, meta-analysis

## Abstract

Background: The effect of probiotic therapy on bacterial vaginosis (BV) is controversial. We conducted a meta-analysis of the efficacy and safety associated with probiotic treatment for BV. Methods: We searched multiple databases covering up to 1 March 2018. Studies published as blinded randomized controlled trials (RCTs), comparing treatment using probiotic versus active or placebo control in BV patients were included, with at least one-month follow-up. Random effects model and trial sequential analysis (TSA) were applied. Results: Ten studies (*n* = 2321) were included. Compared with placebo, the probiotics-only therapy resulted in a beneficial outcome both in clinical cure rate at the 30th day (risk ratio, RR = 2.57; 95% confidential interval, 95% CI: 1.96 to 3.37), and Nugent score (mean difference, MD = −2.71; 95% CI: −3.41 to −2.00). This effect decreased but remained significant after eight weeks. Probiotics-post-antibiotics therapy had a decreased effect only for a short term and possibly among studies with a mostly black study population. No extra adverse events were observed. The TSA suggested a larger sample size for effective evaluation of the probiotics as a supplementary remedy. Conclusions: Probiotic regimes are safe and may exhibit a short-term and long-term beneficial effect for BV treatment. The ethnic-specific result for the probiotic used after antibiotics is worthy of further study.

## 1. Introduction

Bacterial vaginosis (BV) is one of the most common diseases in women of reproductive age [1]. In China, the prevalence of BV ranged from 5.9% to 15.4% [2]. However, this number was 16.3%–29.2% in the United States [3], and reached up to 50% in southern and eastern Africa [4]. Despite this being a mild disease, patients with BV may suffer from a higher risk of reproductive tract infections and adverse pregnancy outcomes [5,6,7]. 

Clinical management of BV has little progress in the past two decades. The first-line antibiotic therapy showed 70%–80% curative rates after four weeks of treatment [1]; however, high rates of recurrence occurred within twelve months, reaching 40%–50% [8]. 

This unsustainable therapeutic impact on BV may limit our understanding of its pathogenesis. For a long time, BV has been regarded as an infectious disease caused by anaerobes, such as *Gardnerella vaginalis*. However, with the advent of culture-independent molecular approaches based on the sequencing of 16S rRNA genes, our understanding of the vaginal microbiome (VMB) has been dramatically improved. Several studies indicated that VMB might play a key role in the development of BV [9]. The occurrence of BV may be associated with the composition and metabolism of certain types of VMB, and probiotics, especially lactobacilli-based, are effective in preventing urogenital diseases [10]. 

Probiotics are “live microorganisms which, when administered in adequate amounts, confer a health benefit on the host” [11]. The use of probiotics for treatment can be dated back to 1907 by Metchnikoff [12], and followed by Newman (1915) and Löser (1920) [13,14]. With the advent of antibiotics during the 1940s, this idea was almost forgotten. However more recently, the interest of this ancient healing technique was rekindled due to the initiation of the Human Microbiome Project, and the number of clinical trials with probiotic therapy has been increasing rapidly [15]. 

Despite the growing clinical evidence, the efficacy of probiotic treatment on BV remains controversial [16,17,18]. Up to now, three meta-analyses have been reported [19,20,21], but none of them has explored the sources of interpretable heterogeneity and its influence on the results. One of them was published in 2009 in the Cochrane Library with only four studies. Another meta-analysis by Huang and colleagues exhibited a considerable heterogeneity (*I^2^* = 87%) of the effect with no further explanations. The latest one, by Tan et al., only focused on the impact of probiotics as a supplementary therapy with metronidazole and attributed the heterogeneity (*I^2^* = 83%) to one single study (the *I^2^* was reduced to 16% after omission of this single study) without further explanations. Thus, a widely accepted consensus has not been formulated yet.

In this paper, we conducted a meta-analysis of all published randomized controlled trials to determine the efficacy and safety of probiotic therapy for BV, explore the potential sources of heterogeneity, and calculate required information size (RIS) for each subgroup using trial sequence analysis (TSA) method.

## 2. Materials and Methods

This study was conducted in accordance with the Preferred Reporting Items for Systematic Reviews and Meta-Analysis (PRISMA) guidelines [22]. The protocol was registered on PROSPERO (CRD42018090057).

### 2.1. Data Sources, Searching Strategy and Eligibility Criteria

We searched Medline, Embase, Cochrane Library, Web of Science, Lilacs, Clinicaltrial.gov and Google Scholar for studies from database inception to 1 March 2018 and reviewed the relevant citations. The full electronic search strategy is shown in Supplementary File 1. We searched the meta-analyses and reviews to find more eligible studies and contacted the investigators if the full-text papers were not available.

Eligibility criteria were based on the PICOS framework [23]: (1) premenopausal nonpregnant women with BV were enrolled (BV was defined by one of three diagnostic tests recommended by widely accepted clinical guidelines [24,25], namely Nugent score [26], Amsel criteria [27] or Hay/Ison criteria [28]); (2) probiotic preparations, single or mixed strains with any dosage, route of administration and preparation types (including capsules, tablets, tampons, pessaries, and effervescent tablets); (3) control arm used placebo or active controls (with an active ingredient including metronidazole, tinidazole, and clindamycin); (4) the outcomes of interest were reported (see Section 2.3); (5) randomized, double- or triple-blind controlled trials with follow-up of at least one menstrual cycle (verification time of BV cure according to guidelines from the FDA [29]). 

The following criteria determined the ineligible studies: (1) unsuitable participants according to the diagnostic tests (e.g., prophylaxis for BV); (2) preparations without living bacterium, and food intake as probiotic preparations (e.g., yogurts); (3) offering a more aggressive treatment of BV in control group than routine use in standard clinical practice (e.g., much longer duration and higher dose, treatment of sexual partners); (4) less than 20 patients; (5) reported in neither English nor Chinese; (6) retracted or plagiarized papers; (7) inappropriate study designs, non-blind methods or non-RCTs; (8) full-text papers were not available; (9) studies with pregnant women. 

### 2.2. Data Extraction and Bias Assessment

The information on the literature search and the inclusion/exclusion criteria set was extracted independently by two investigators (Wang and He) according to a standard protocol (see Supplementary Files 2–4). All data were stored in Microsoft Excel 2016 (Microsoft Corporation, Washington, USA). We contacted 19 authors by e-mail to request additional data and information in 11 studies, including demographic and behavioral characteristics of the enrolled participants, efficacy and safety outcomes, statistical principles and strategies to prevent biases. Four authors (C. Gille and C.F. Poets [30], P.G. Larsson [31], R.C. Martinez [16]) presented or confirmed the data, two authors (P.G. Larsson, R.C. Martinez) presented the details of statistical issues, and one author (R.C. Martinez) clarified the design for bias prevention.

The risk of bias was assessed with the Cochrane Collaboration’s tool, recommended by the Cochrane Collaboration for randomized trials. This assessment was done at the study level. Data extraction and risk estimates were performed by two investigators. Disagreements were resolved by consensus with Zheng, the principal investigator. 

### 2.3. Outcomes

Primary outcomes were as follows: clinical cure rates measured after a regular menstrual cycle (short-term, typically at the 28th/30th day after intervention) or more than two menstrual cycles (long-term, usually more than 60 days) by an absence of Amsel criteria, a Nugent score <4 [27] or the Hay/Ison classification grade 1 [32]; Nugent score (at the same time as the clinical cure rate). 

Secondary outcomes were as follows: incidence rates of adverse events (AE, defined as any untoward medical occurrence associated with the use of a drug, whether or not being considered drug-related [33]). 

### 2.4. Statistical Analysis

#### 2.4.1. Synthesis of Results

The analysis of the treatment effect at different times were done separately with an intention-to-treat (ITT) approach. We expressed dichotomous data (clinical cure rate, incidence rates of AE) as risk ratios (RR) or risk differences (RD) with 95% confidence intervals (95% CI), and continuous data (Nugent score) as mean differences (MD) with 95% CI. When different measures were used to assess the same continuous outcome, standardized mean differences (SMD) with 95% CI were calculated. 

The summary estimates above were calculated using the random effects model (the Mantel–Haenszel method for dichotomous outcomes, and the inverse variance method for continuous outcomes). The non-continuity correction was used for trials with zero events to enable finite variance estimators’ availability. Publication bias was assessed by Egger’s test (two-tailed *p* < 0.1 considered to be asymmetry).

#### 2.4.2. Identification of Heterogeneity

We assessed heterogeneity using Cochran’s Q test [33] and Higgins’ *I^2^* statistic [34] for each meta-analysis. Higgins’ *I^2^* was used to classify the heterogeneity according to the reference (<25% = low; 25%–50% = moderate; 50%–75% = substantial; >75% = high). For moderate to high heterogeneity, several pre-specified subgroup analyses were conducted to determine possible reasons including patients’ characteristics (age, race, multiple/new sex partners, lack of condom use, vaginal douching, tobacco/alcohol use) and design of the intervention (probiotic strains, form, route, total intake, additional interventions) which may introduce clinical heterogeneity. Particularly, we divide the race into two groups: the white-dominant group (the proportion of Caucasian participants higher than 50%), and the mixed ethnic group (mostly black women, the proportion of Caucasian participants lower than 50%). We also performed meta-regression analyses if feasible (>10 studies available in one analysis). If both of these methods failed, we would attempt to find the causes of heterogeneity by examining any individual study.

#### 2.4.3. Additional Analyses

Since some quite strict eligibility criteria were set, we would relax them one by one in sensitivity analyses, with other factors being equal. New RCTs with the following characteristics were included: studies with abstracts only, with food or dietary supplements as interventions, with a high risk of bias, less than 20 participants, or with non-blind outcome assessment. We re-evaluated the overall effects to see there was any change. We also investigated attrition, such as dropouts, loss to follow-up and withdrawals. No outcomes of efficacy or safety were imputed according to the principle of analysis.

Most meta-analyses contain a limited number of trials, so findings of interventional effects could be subject to random error or lack of statistical power (type II error). We incorporated TSA to adjust for random error risks and provide the RIS to solve these problems [35].

Statistical analyses in meta-analyses were done with Stata (Version 14.0; StataCorp LLC, College Station, TX, USA) and Review Manager (Version 5.3; Copenhagen: the Nordic Cochrane Centre, the Cochrane Collaboration, Copenhagen, Denmark, 2014). The trial sequential analysis model was run in TSA (Version 0.9.5.5 Beta; Copenhagen Trial Unit, Copenhagen, Denmark).

## 3. Results

### 3.1. Study Selection and Characteristics

**Figure 1:**
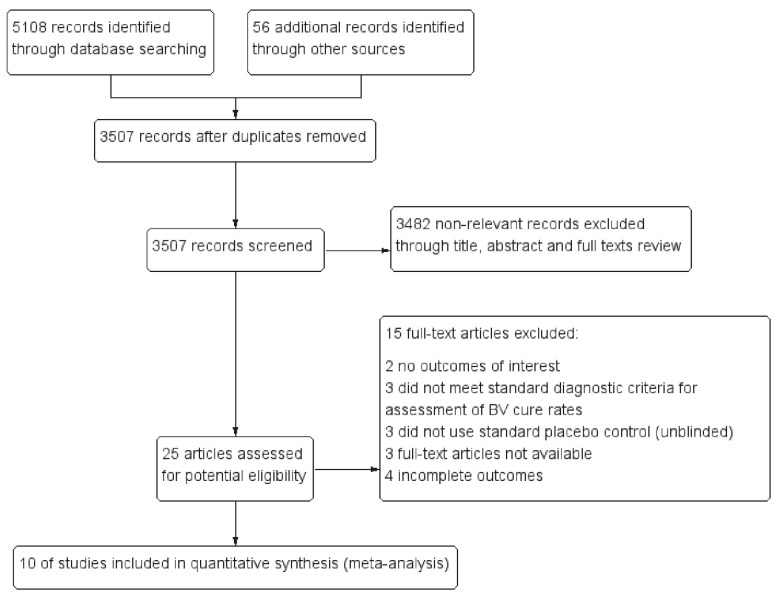
Study flow diagram. Abbreviations: RCTs = randomized controlled trials; BV = bacterial vaginosis.

After a screening of more than 5000 bibliographic references, only ten RCTs (*n* = 2321) [16,17,18,31,36,37,38,39,40,41] were available for analysis. Those trials were all placebo-controlled, one out of them was both active- and placebo-controlled (active comparison groups were treated with vaginal clindamycin). Participants in seven studies were firstly treated with antibiotics then followed with probiotic preparations to reduce the risk of recurrence; the other three studies used probiotics only. Figure 1 shows the flow chart of the study selection. Table S1 lists the main characteristics of the included studies. Supplementary Files 2–4 provide more information.

### 3.2. Risk of Bias within/across Studies

Figures S1 and S2 summarize the potential sources of bias for those included studies at the study level, assessed by the Cochrane Collaboration’s tool. Most of the studies were classified as “low” or “unclear” risk. Table S2 lists the detailed information of risk of bias within each of them.

### 3.3. Synthesis of Results

#### 3.3.1. Efficacy: Negative Control (Placebo)

##### Short-Term Effect: after a Normal Menstrual Cycle

**Figure 2:**
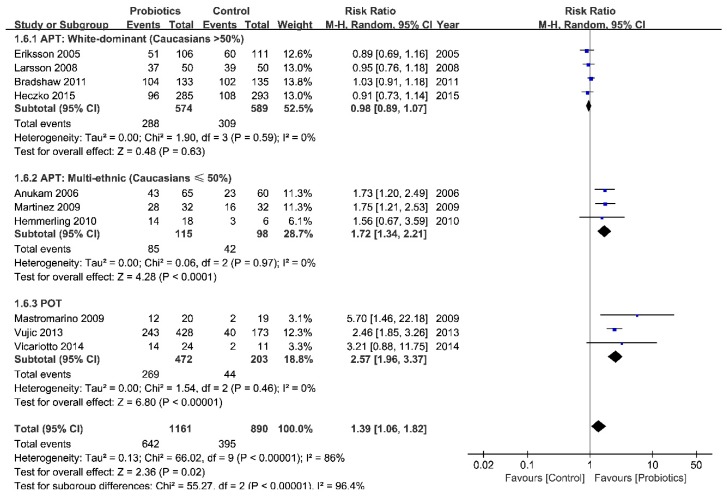
Forest plot: the efficacy of probiotics vs. placebo for BV after a normal menstrual cycle (around the 30th day after intervention, divided by ethnic groups). Abbreviations: APT = antibiotic + probiotic treatment; POT = probiotic only treatment; NA = no data available.

With data from 2047 patients in 10 studies, we divided studies into two categories: probiotic-only therapy [POT] and antibiotic plus probiotic combination therapy [APT]. The meta-analysis showed a statistically significant benefit from POT compared with placebo (pooled RR from 675 patients in three trials = 2.57 (95% CI = 1.96 to 3.37)), but not with APT (pooled RR from 1376 patients in seven trials = 1.11 (95% CI = 0.94 to 1.31)). Nevertheless, a moderate statistical heterogeneity still appeared in APT (*I^2^* = 69%, Figure S3). Based on the results of subgroup analysis, we found evidence that treatment with probiotics benefited those patients in the mixed ethnic group (pooled RR from 213 patients in three trials = 1.72 (95 % CI = 1.34 to 2.21), *I^2^* = 0%), but did not find such evidence for the white-dominant group (pooled RR from 1163 patients in 4 trials = 0.98 (95% CI = 0.89 to 1.07), *I^2^* = 0%) (Figure 2). Although we did all the subgroup analyses mentioned previously, there was no statistical significance for all characteristics except for ethnic group and antibiotic usage (Table 1).

**Figure 3:**
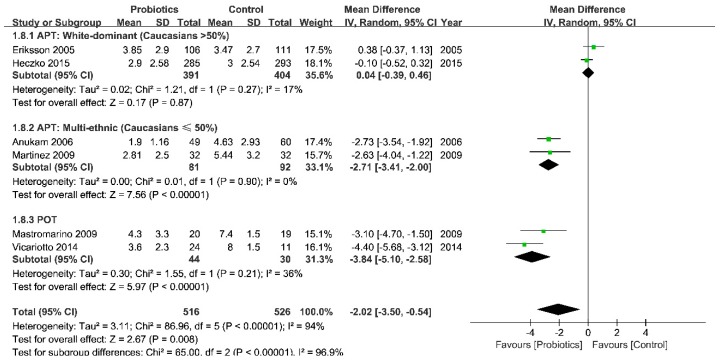
Forest plot: Nugent scores of probiotics vs. placebo for BV after a normal menstrual cycle (around the 30th day after intervention, divided by ethnic groups). Abbreviations: APT = antibiotic + probiotic treatment; POT = probiotic only treatment; NA = no data available.

A significant reduction in Nugent scores around the 30th day was also seen in POT (pooled mean difference (MD) from 74 patients in two studies = −3.84 (95% CI = -5.10 to −2.58)), but not in APT (pooled MD from 968 patients in four studies = −1.20 (95% CI = −2.68 to 0.27)). Consistent with the subgroup analysis in clinical cure rate, the Nugent score did not change much in white-dominant group (pooled MD from 795 patients in two studies = 0.04 (95% CI = −0.39 to 0.46)), but it improved markedly in mixed ethnic subgroup (pooled MD from 173 patients in two studies = −2.71 (95% CI = −3.41 to −2.00)) (Figure 3).

##### Long-Term Effect: More Than Two Menstrual Cycles

We enrolled six studies (*n* = 1824) in this analysis. After eight weeks of treatment, POT was still associated with a more favorable clinical response (pooled RR from 636 patients in two trials = 1.58 (95% CI = 1.24 to 2.01)), though it was lower than the RR in the short-term trials. However, the effect of APT was similar to those treated with placebo (pooled RR from 1188 patients in four trials = 0.97 (95% CI = 0.84 to 1.11)). Additionally, there was no evidence of an interaction between time and treatment effect (test of subgroup difference: *Z* = 1.80, *p* = 0.07, Figure S4).

#### 3.3.2. Efficacy: Positive Control (Antibiotics)

We did not find any end-to-end comparisons of probiotic-only therapy and antimicrobial-only therapy in eligible trials by comprehensive literature searching.

#### 3.3.3. Safety

Patients in the probiotic group did not show more adverse events compared with placebo group (pooled RD from 1415 patients in 10 studies = 0.01 (95% CI = −0.02 to 0.04)). Additional analysis was performed with adverse events on the gastrointestinal tract and genitourinary system (including superinfection, like vulvovaginal candidiasis). No adverse events were considered to be drug-related (Figure S5, Table S3).

### 3.4. Additional Analysis

#### 3.4.1. Publication Bias and Selective Reporting

The funnel plot on the short-term clinical cure rate was considered to be asymmetrical by Egger’s test (t = 2.62, *p* = 0.030, Figure S6). Two small studies from POT had larger effect sizes. We tried the trim-and-fill method to make the funnel plot symmetrical. However, the results from further analysis were accordant with those previous (t = 1.871, *p* = 0.061). This result raises the inevitable possibility of publication bias.

#### 3.4.2. Sensitivity Analyses

We added new RCTs in sensitivity analyses to re-evaluate the overall effects (criterion was described previously in “Section 2.4.3”), and all results were consistent with our main findings (Table S4) [30,42,43,44,45,46]. There were no significant small-study effects in all analyses by iteratively removing one study at a time (Figures S7–S9). Using the methods of imputation to deal with missing data may change the estimate of several outcomes of efficacy, but the direction of effect remains unchanged in subgroup analyses (Table S5). The results had little uncertainties in general.

#### 3.4.3. Trial Sequential Analysis

We used TSA to evaluate whether the comparisons had adequate power in both APT and POT groups. POT has surpassed its RIS (RIS = 655, actual sample size 675), the cumulative Z curve for POT versus placebo had crossed both the conventional boundary and the trial sequential monitoring boundary (*Z* = −7.93, TSA monitoring boundaries = −1.99, α = 0.05, β = 0.1; relative risk reduction, RRR = 20%), which meant the response of POT was truly positive. Although the reaction of APT was negative, the sample size was far smaller than its required information size (APT: RIS = 3473, actual sample size 1376), which suggested that more multicenter randomized controlled trials with larger sample sizes are needed (Figures S10 and S11).

## 4. Discussion

This meta-analysis involved 10 RCTs with a low or moderate risk of bias, which suggested that the treatment with probiotics alone was more effective in the therapy of BV for both short- and long-term; however, the probiotics used after antibiotic treatment was effective only for a short term.

Treatment of BV with currently recommended antibiotics has always been in great dispute with its high rates of recurrence. Two possible mechanisms might explain these results. Firstly, the BV-associated bacteria and multispecies BV biofilm may be involved in the potential pathological pathway of developing such disease [47]. *G. vaginalis*, for example, was found to have activated genes that could repair the DNA damage caused by the antibiotics, which may lead to a high recurrence rate after therapy [48]. Therefore, the use of probiotics was meant to consolidate the efficacy of the antibiotic and prevent BV recurrence. Secondly, compared with the Caucasian women, black women are likely to have a more diverse vaginal microbial profile [49], higher prevalence of BV and Nugent scores, and a much stronger immune response related to BV-associated bacteria [50]. Conversely, the vaginal flora of Caucasian women was mostly dominated by *Lactobacillus*. Specific probiotics may even inhibit the indigenous microbiome and induce a delayed post-antibiotic reconstitution [51]. Hence the introduction of lactobacilli after antibiotic treatment may not be useful in rebuilding a solid micro-ecosystem. These fundamentally racial differences may introduce the heterogeneity [9].

In contrast, probiotic therapy alone showed a better benefit in efficacy for BV treatment over the placebo. Since the products (H_2_O_2_ and lactic acid) produced by the lactobacilli have been found to inhibit the growth of BV-associated bacteria [52], we surmised the extraneous lactobacilli might gradually colonize the vaginal flora through competing. TSA analysis further indicated its promising clinical application. Given all that, the first-line therapy of antibiotics seemed unnecessary by contrast with the effective probiotics-only therapy.

Our study is the first one to identify the possible sources of heterogeneity in the efficacy of probiotics treatment for BV and to use TSA to improve the validity of evidence. Besides, we separated the short-term from the long-term effects of probiotic preparations and pooled outcomes with a quite similar follow-up time point. Nonetheless, there are some limitations. Firstly, we identified a possible ethnic-specific efficacy for APT only through a few clinical trials, we could not separate the effect of either of the races alone. However, it should be emphasized that we obtained highly consistent results in both primary efficacy outcomes (clinical cure rates and Nugent scores), which could strengthen the power of our conclusions. Secondly, the strength of the results of meta-regression with subgroups analysis we performed may be weak with the low number of studies identified [53,54]. Thirdly, without any data on the prevalent microbial population, there could be a significant difference in the virginal microbiomes among the BV patients in our study [9,55]. Fourthly, we have no idea about the effects of probiotics in Asian populations due to limited studies. Fifthly, based on the results from TSA and etiological study, sample sizes of several outcome measures were far less than its RIS. Last but not least, we were not able to neglect the publication bias.

## 5. Conclusions

In conclusion, probiotic-only regimes are safe and may exhibit short-term and long-term beneficial effects for BV treatment. The ethnic-specific result for the probiotics used after antibiotics is worthy of further study.

## Figures and Tables

**Table 1:** Summary of subgroup analysis results.

Groups	No. of Studies	*n* Total	RR (95% CI)	*p* (Overall Effect)	*I^2^*, %	*p* (Heterogeneity)
All studies	10	2047	1.39 (1.05 to 1.83)	0.02	90	<0.00001
Type of intervention						
APT	7	1376	1.11 (0.94 to 1.31)	0.22	69	0.004
POT	3	675	2.57 (1.96 to 3.37)	<0.00001	0	0.46
Diagnostic Standard (APT)						
Amsel	5	983	1.07 (0.85 to 1.35)	0.58	65	0.02
Nugent	2	393	1.30 (0.76 to 2.22)	0.33	87	0.006
Ethics of participants (APT)						
White-dominant	4	1163	0.98 (0.89 to 1.07)	0.63	0	0.59
Multi-ethnic	3	213	1.72 (1.34 to 2.21)	<0.0001	0	0.97
Route of intervention (APT)						
Vaginally	4	609	1.00 (0.90 to 1.11)	0.97	0	0.50
Orally	3	767	1.38 (0.85 to 2.23)	0.19	86	0.0009
Number of strains (APT)						
1	2	292	1.05 (0.87 to 1.27)	0.59	8	0.30
2	3	289	1.39 (0.87 to 2.23)	0.17	85	0.001
3	2	795	0.92 (0.77 to 1.09)	0.32	0	0.97
Dosage per capsule (APT)						
≤1 × 10^8^ CFU	2	485	1.01 (0.91 to 1.13)	0.81	20	0.26
(1–10) × 10^8^ CFU	3	803	1.11 (0.80 to 1.53)	0.54	79	0.009
>10 × 10^8^ CFU	2	88	1.72 (1.22 to 2.41)	0.002	0	0.80
Dosage in total (APT)						
≤1 × 10^9^ CFU	2	485	1.00 (0.91 to 1.10)	0.94	20	0.26
(1–10) × 10^9^ CFU	2	703	1.06 (0.88 to 1.28)	0.53	88	0.003
>10 × 10^9^ CFU	3	188	1.21 (1.00 to 1.46)	0.05	77	0.01

Abbreviations: APT = antibiotic + probiotic treatment; POT = probiotic only treatment; CFU = colony-forming units.

## Supplementary Materials

The following are available online at https://www.mdpi.com/1660-4601/16/20/3859/s1, Table S1: Characteristics of 10 eligible studies, Table S2: Risk of bias source of 10 studies, Table S3: Summary of safety analysis results, Table S4: Summary of sensitivity analysis results, Table S5: Attrition analysis of Efficacy on Day 30, Supplementary file 1: Search Strategy, Supplementary file 2: Standardized form of data extraction—basic information & participants, Supplementary file 3: Standardized form of data extraction—intervention, Supplementary file 4: Standardized form of data extraction—the outcome, Supplementary file 5: Other sensitivity analysis, Figure S1: Risk of bias graph, Figure S2: Risk of bias summary, Figure S3: Forest plot: the efficacy of probiotics vs. placebo for BV after a normal menstrual cycle (around the 30th day after intervention, divided by different intervention groups), Figure S4: Forest plot: the efficacy of probiotics vs. placebo for BV after two menstrual cycles or later (divided by ethnic groups), Figure S5: Safety analysis, Figure S6: Funnel plot on the efficacy of probiotics treatment for BV around 30th day, Figure S7: Sensitivity Analysis: Remove-one analysis of all subgroup, Figure S8: Sensitivity Analysis: Remove-one analysis of APT group, Figure S9: Sensitivity Analysis: Remove-one analysis of POT group, Figure S10: The cumulative Z—curves of TSA: Efficacy after a regular menstrual cycle)around the 30th day after intervention), Figure S11: The cumulative Z—curves of TSA: Efficacy after a regular menstrual cycle (around the 30th day after intervention, Probiotics vs. Placebo).
