# Supplementary material for: Probiotics for the Treatment of Bacterial Vaginosis: A Meta-Analysis
Source: Int J Environ Res Public Health. 2019 Oct 12;16(20):3859. doi: 10.3390/ijerph16203859 (PMC6848925; doi:10.3390/ijerph16203859)
Supplement: Supplementary file 1 [file ijerph-16-03859-s001.zip › Supplementary files/Supplementary file 1 - search strategy.docx]

**Supplementary file 1** Search Strategy

| **Database** | **Search Strategy** |
| --- | --- |
| MEDLINE | (((probiotic*[Title/Abstract] OR lactobacill*[Title/Abstract] OR bifidobacteri*[Title/Abstract] OR "lactic acid bacteria"[Title/Abstract]))) AND (((Vaginitides[Title/Abstract] OR Vaginosis[Title/Abstract] OR Vaginitis[Title/Abstract] OR Vaginoses[Title/Abstract] OR Vaginities[Title/Abstract] OR vaginale[Title/Abstract] OR colpitis[Title/Abstract])) AND (Bacterial[Title/Abstract] OR bacteria[Title/Abstract] OR bacterium[Title/Abstract] OR nonspecific[Title/Abstract] OR “non specific”[Title/Abstract] OR non-specific[Title/Abstract] OR unspecific[Title/Abstract] OR Gardnerella[Title/Abstract] OR anaerobic[Title/Abstract] OR Corynebacterium[Title/Abstract] OR hemophilus[Title/Abstract] OR Aerobic[Title/Abstract] OR Amine[Title/Abstract])) |
| EMBASE | #1 'vaginitides':ab,ti OR 'vaginosis':ab,ti OR 'vaginitis':ab,ti OR 'vaginoses':ab,ti OR 'vaginities':ab,ti OR 'vaginale':ab,ti OR 'colpitis':ab,ti  #2 'bacterial':ab,ti OR 'bacteria':ab,ti OR 'bacterium':ab,ti OR 'nonspecific':ab,ti OR 'non specific':ab,ti OR 'non-specific':ab,ti OR 'unspecific':ab,ti OR 'gardnerella':ab,ti OR 'anaerobic':ab,ti OR 'corynebacterium':ab,ti OR 'hemophilus':ab,ti OR 'aerobic':ab,ti OR 'amine':ab,ti  #3 'probiotic*':ab,ti OR 'lactobacill*':ab,ti OR 'bifidobacteri*':ab,ti OR 'lactic acid bacteria':ab,ti  #4 #1 AND #2 AND #3 |
| Cochrane Library | '((Vaginitides or Vaginosis or Vaginitis or Vaginoses or Vaginities or vaginale or colpitis) and (Bacterial or bacteria or bacterium or nonspecific or non specific or non-specific or unspecific or Gardnerella or anaerobic or Corynebacterium or hemophilus or Aerobic or Amine) and (probiotic* or lactobacill* or bifidobacteri* or lactic acid bacteria)) |
| Web of Science | #1 TS=(Vaginitides or Vaginosis or Vaginitis or Vaginoses or Vaginities or vaginale or colpitis)  #2 TS= (Bacterial or bacteria or bacterium or nonspecific or non specific or non-specific or unspecific or Gardnerella or anaerobic or Corynebacterium or hemophilus or Aerobic or Amine)  #3 TS= (probiotic* or lactobacill* or bifidobacteri* or lactic acid bacteria)  #4 #1 and #2 and #3 |
| LILACS | Vaginitides OR Vaginosis OR Vaginitis OR Vaginoses OR Vaginities OR vaginale OR colpitis [Abstract words] and Bacterial or bacteria or bacterium or nonspecific or non specific or non-specific or unspecific or Gardnerella or anaerobic or Corynebacterium or hemophilus or Aerobic or Amine [Abstract words] and probiotic$ or lactobacill$ or bifidobacteri$ or lactic acid bacteria [Abstract words] |
| Google Scholar | allintitle: (probiotic OR lactobacilli OR lactobacillus OR "lactic acid bacteria") AND (Vaginitides OR Vaginosis OR Vaginitis OR vaginale OR colpitis) AND (Bacterial OR bacteria OR nonspecific OR unspecific OR Gardnerella OR anaerobic OR Aerobic) |
| China National Knowledge Infrastructure (CNKI) | 检索条件：发表时间 between (1900-1-1,2016-09-01) 并且 ( ( ( 主题=同义词扩展(双歧杆菌) 或者 主题=同义词扩展(益生菌) ) 或者 ( 主题=同义词扩展(乳酸菌) 或者 主题=同义词扩展(乳杆菌) ) ) 或者 主题=同义词扩展(合生元) ) 并且 ( ( ( ( 主题=同义词扩展(细菌性阴道病) 或者 主题=同义词扩展(细菌性阴道炎) ) 或者 ( 主题=同义词扩展(非特异性阴道炎) 或者 主题=同义词扩展(需氧性阴道炎) ) ) 或者 ( 主题=同义词扩展(棒状杆菌性阴道炎) 或者 主题=同义词扩展(嗜血杆菌性阴道炎) ) ) 或者 ( 主题=同义词扩展(厌氧性阴道炎) 或者 主题=同义词扩展(加德菌性阴道炎) ) ) 并且 发表时间 between (1900-1-1,2016-09-01) 并且 ( ( ( 主题=同义词扩展(治疗) 或者 主题=同义词扩展(疗法) ) 或者 ( 主题=同义词扩展(临床) 或者 主题=同义词扩展(试验) ) ) 或者 ( 主题=同义词扩展(疗效) 或者 主题=同义词扩展(评价) ) ) (模糊匹配),专辑导航：全部; 数据库：文献 跨库检索 |
| Clinicaltrial.gov | probiotic \| Vaginosis, Bacterial |
| Chinese BioMedical Disc (CBM Disc) | #1 (((((((("细菌性阴道病"[常用字段] OR "细菌性阴道炎"[常用字段] OR "非特异性阴道炎"[常用字段] OR "阴道病, 细菌性"[主题词]) OR "细菌性阴道炎"[常用字段]) OR "非特异性阴道炎"[常用字段]) OR "需氧性阴道炎"[常用字段]) OR "棒状杆菌性阴道炎"[常用字段]) OR "嗜血杆菌性阴道炎"[常用字段]) OR "厌氧性阴道炎"[常用字段]) OR "加德菌性阴道炎"[常用字段]) AND -2016[日期]  #2 ((((((("双歧杆菌"[常用字段]) OR "益生菌"[常用字段]) OR "乳酸菌"[常用字段]) OR "乳杆菌"[常用字段]) OR "合生元"[常用字段]) OR "微生态制剂"[常用字段]) OR "活菌"[常用字段]) AND -2016[日期]  #3 (((((("治疗"[常用字段]) OR "疗法"[常用字段]) OR "临床"[常用字段]) OR "试验"[常用字段]) OR "疗效"[常用字段]) OR "评价"[常用字段]) AND -2016[日期]  #4 (#1) AND (#2) AND (#3) |
| Wanfang Database | 摘要:((细菌性阴道病 OR 细菌性阴道炎 OR 非特异性阴道炎 OR 需氧性阴道炎 OR 棒状杆菌性阴道炎 OR 嗜血杆菌性阴道炎 OR 厌氧性阴道炎 OR 加德菌性阴道炎)) * 摘要:((治疗 OR 疗法 OR 临床 OR 试验 OR 疗效 OR 评价)) * 摘要:((双歧杆菌 OR 益生菌 OR 乳酸菌 OR 乳杆菌 OR 合生元)) * Date:-2016 |
