# Supplementary material for: Probiotics for the Treatment of Bacterial Vaginosis: A Meta-Analysis
Source: Int J Environ Res Public Health. 2019 Oct 12;16(20):3859. doi: 10.3390/ijerph16203859 (PMC6848925; doi:10.3390/ijerph16203859)
Supplement: Supplementary file 1 [file ijerph-16-03859-s001.zip › Supplementary files/Supplementary file 2 - standardized form - basic information.docx]

**Supplementary file 2** Standardized form of data extraction – Basic information & participants

| **No.** | **Basic information** | **Study design** | **Participants** | | | | | |  |  |
| --- | --- | --- | --- | --- | --- | --- | --- | --- | --- | --- |
|  | **Author, publication year, study site, country** |  | **No. age range (mean/ median), JS** | **Ethnic group** | **Inclusion** | **Exclusion** | **Diagnostic standards of BV [1]** | **Sex life** | **Contraceptive** | **Alcohol & tobacco usage** |
| 1 | Eriksson 2005  Finland, Norway & Sweden | Multicentre randomized, double blind, placebo-controlled trial | 255, >18 years (32), 4 | Caucasian 178, Other 9, Lost or excluded 68 | 1. Premenopausal women > 18 years old, diagnosed with BV.  2. Willing to use tampons during menstruation period, signed informed consent. | 1. Pregnant or planning for pregnancy or lactation.  2. Ongoing yeast/Chlamydia trachomatis infection.  3. Undergoing antibiotic treatment during the week preceding the diagnosis. | Amsel's criteria | No data available | 48 patients used contraceptive pills, 53 used IUDs, 26 for condoms and 60 for other methods. | No data available |
| 2 | Anukam 2006a  Benin City, Nigeria | Randomized, double blind, placebo-controlled trial | 125, 18—44 years (NA), 7 | Black | 1. Women with symptomatic BV.  2. Signed informed consent. | 1. Pregnancy, lactation & menstruation at time of diagnosis.  2. Evidence of gross inflammatory genital processes, including any sexually transmitted infection (including HIV/AIDS).  3. Use of systemic or intravaginal antibacterial agents within the previous 14 days.  4. Use of investigational drugs within 30 days.  5. Allergy to metronidazole, warfarin, lithium or disulfiram. | Nugent's criteria & BV Blue test | No data available | 21 patients used oral pills, 28 for condoms, 7 for IUDs. | No data available |
| 3 | Larsson 2008  Drammen, Norway | Randomized, double-blind, placebo-controlled trial | 100, 18.8—53.6 years (34.3), 6 | NA | 1. Women >18 years old with symptomatic BV.  2. Regularly menstruating with normal gynecological status.  3. Signed informed consent. | 1. Pregnancy or lactation.  2. Hormonal IUD and signs of other genital tract infections (Candida infection or Trichomonas vaginalis). | Amsel’s criteria | No data available | Users of hormonal IUD were excluded | No data available |
| 4 | Martinez 2009  São Paulo, Brazil | Multicentre randomized, double blind, placebo-controlled trial | 64, NA (30.2), 5 | 60% Black/ mulatto, 40% Caucasian | Women diagnosed with BV, signed informed consent. | 1. Immunosuppress.  2. Diagnosis of VVC or trichomoniasis.  3. Use of systemic or intravaginal antibacterial agents currently or within the previous 14 days.  4. Hypersensitivity to imidazoles.  5. Menstruation at time of diagnosis. | Nugent's & Asmel's criteria | No data available | 40 patients used contraceptive methods, 21 in treatment arm and 19 in control arm. | No data available |
| 5 | Mastromarino 2009  Rome, Italy | Randomized, double-blind, placebo-controlled trial | 39, 18—NA years (34), 7 | NA | 1. Premenopausal women >18 years old, With a known history of recurrent BV.  2. Signed informed consent. | 1. Pregnancy.  2.Diabetes,  3. Use of antibiotics or vaginal antimicrobials in the previous 14 days.  4. Trichomonas vaginalis, yeast or N. gonorrhoeae infection. | Asmel’s criteria. | No data available | No data available | No data available |
| 6 | Hemmerling 2010  San Francisco, USA | Randomized, double-blind, placebo-controlled trial, phase 2a | 24,18—50 years (29.5), 6 | 12 Caucasians, 9 black, 5 others | 1.Premenopausal women between 18—50 years of age, diagnosed with BV.  2. Signed informed consent. | 1. Pregnant, within 2 months after pregnancy or lactation.  2. Genital epithelial disruption or abnormal cervical cytology.  3.Urinary tract infection & STD, including HIV, recurrent genital herpes, syphilis, vaginal candidiasis, Trichomonas vaginalis, Neisseria gonorrhoeae, or Chlamydia trachomatis.  4. New long-acting contraception in the past 2 months.  5. History of recurrent vaginal infections (≥2 in past 6 months).  6. Active uncontrolled medical condition.  7. Use of another probiotics product or new investigational drug within 30 days; or an immunosuppressive drug within 60 days. | Nugent's & Asmel's criteria | No data available | Users of new long-acting contraception in the past 2 months were excluded | No data available |
| 7 | Bradshaw 2011/2012  Melbourne, Australia | Randomized double-blind placebo/antibiotic controlled trial | 450, 18-49 years (27), 6 | 312 Australian/British, 136 others, 2 NA. | 1. Women aged between 18 and 50 with symptomatic BV.  2. Signed informed consents. | 1. HIV positive.  2. Pregnancy, lactation or attempting to conceive.  3. Not fluent in English, without an Australian-postal address.  4. Not able to abstain from vaginal sex during vaginal therapy if they were reliant on 100% condom use for STI protection/contraception. | Nugent's or Asmel's criteria | 94 patients had regular sexual partners in treatment arm, compared to 112/96 in positive/negative control arm. | 146 patients used contraceptive methods, 50 in treatment arm, 54 in positive control arm and in 42 negative control arms. | 75 smokers in treatment arm, compared to 62/55 in positive/negative control arm. |
| 8 | Vujic 2013  Central and northwestern Croatia | Multicentre randomized, double-blind,  placebo-controlled trial | 651,18-58 years (33.0), 6 | NA | 1. Women >18 years old, diagnosed with BV.  2. Signed informed consent. | Pregnancy, lactation, menstruation. | Nugent's or Asmel's criteria | 74.50%/70.89% had <3 sex partners in treatment/control arm | 16.78%/28.41% used condom, 24.83%/20.81% used oral contraceptives and 8.05%/8.01% used IUD in treatment/control arm | 68.46%/64.56% non-smokers, 56.38%/50.07% non-drinkers in treatment/control arm |
| 9 | Vicariotto 2014  Milan, Italy | Randomized, double-blind placebo-controlled trial | 35, 18-50 years (34.7), 4 | NA | 1. Women >18 years old with symptomatic BV.  2. Signed informed consent. | 1. Pregnancy.  2. Mixed infections or HIV positive.  3. Treatment with antibiotic agents or other products containing lactobacilli or bifido-bacteria (even if taken orally) in the previous 2 months. | Nugent's or Asmel's criteria | No data available | No data available | No data available |
| 10 | Heczko 2015  Krakow & Warsaw, Poland | Multicentre randomized, double-blind,  placebo-controlled trial | 578, 18-50 years (NA), 6 | Caucasians | 1. Women aged between 18 and 50, diagnosed with BV.  2. Signed informed consent. | 1. Pregnant or breastfeeding.  2. Hypersensitivity to the investigated product, metronidazole or other antibiotics.  3. Candida vaginitis, bleeding from the genital tract of unknown aetiology, any pathology of the reproductive organs.  4. Congenital or acquired immunodeficiency, diabetes, mental illness, or neoplastic disease.  5. Used mechanical contraceptives (diaphragms, intrauterine contraceptive inserts, or hormonal vaginal rings).  6. Used oral hormonal preparations or vaginal oestrogens.  7. Using another oral/vaginal probiotics at the time of assessment for inclusion.  8. Participated in another clinical study within the previous 30 days.  9. Receiving antibiotic therapy for another reason.  10. Scheduled for surgery or hospitalization. | Asmel's criteria | 270/266 patients had regular sexual partners in treatment/control arm | Users of oral hormonal preparations or vaginal estrogens were excluded. 100 patients used contraceptive methods and 55 in treatment arm and 45 in control arm. | 46 smokers in treatment arm, compared to 32 in control arm. |

[1] A. Nugent score - This is used as a gold standard for studies and relies upon estimating the relative proportions of bacterial morphotypes on a Gram stained vaginal smear to give a score between 0 and 10. A score of <4 is normal, 4-6 is intermediate and >6 is BV.

B. Hay Ison criteria - Grade 0: Not related to BV, epithelial cells only, no lactobacilli, indicates recent antibiotics. Grade 1: (Normal): Lactobacillus morphotypes predominate. Grade 2: (Intermediate): Mixed flora with some lactobacilli present, but Gardnerella or Mobiluncus morphotypes also present. Grade 3: (BV): Predominantly Gardnerella and/or Mobiluncus morphotypes, clue cells. Few or absent Lactobacilli. Grade 4: Not related to BV, Gram +ve cocci only, no lactobacilli (Aerobic vaginitis flora)

C. Amsel criteria - The presence of three of the 4 criteria is required; as three are clinical criteria it is possible to make a diagnosis of BV without microscopy or the use of a microbiology laboratory. 1. Homogeneous grey-white discharge. 2. pH of vaginal fluid > 4.5 (measured using narrow gauge pH paper). 3. Fishy odour (if not recognizable, use few drops of 10% KOH). 4. Clue cells present on wet mount microscopy (>20% of all epithelial cells)
