# Supplementary material for: Probiotics for the Treatment of Bacterial Vaginosis: A Meta-Analysis
Source: Int J Environ Res Public Health. 2019 Oct 12;16(20):3859. doi: 10.3390/ijerph16203859 (PMC6848925; doi:10.3390/ijerph16203859)
Supplement: Supplementary file 1 [file ijerph-16-03859-s001.zip › Supplementary files/Supplementary file 3 - standardized form - intervention.docx]

**Supplementary file 3** Standardized form of data extraction – Intervention

| **No.** | | **Basic information** | **Intervention** | | | | | | |
| --- | --- | --- | --- | --- | --- | --- | --- | --- | --- |
|  |  | **Author, publication year, study site, country** | **Probiotics group (No.)** | **Control group (No.)** | **Probiotics Species/Strains** | **Dosage & form** | **Route** | **Duration** | **Follow-up time** |
| 1 | Eriksson 2005  Finland, Norway & Sweden | | First treated with Dalacin® 100 mg clindamycin ovules vaginally once daily for 3 days, manufactured by Pharmacia Upjohn, Stockholm, Sweden. Use tampons during the following menstruation impregnated with lactobacilli (Medipharm AB, Kågeröd, Sweden), mixed with coconut fat (Karlshamns AB, Karlshamn, Sweden). (127). | First treated with same clindamycin ovules. During the following menstruation, use placebo tampons (128). | Lactobacillus gasseri, Lactobacillus casei var rhamnosus & Lactobacillus fermentum | Tampon, contained 108 living lactobacilli per tampon, >5 tampons during menstruation. | Vaginally | Depending on the length of menstruation. | 2 follow-up visits in 2 menstrual cycles (≈60 days in total, After the 1st and the 2nd menstruation). |
| 2 | Anukam 2006a  Benin City, Nigeria | | Treated with 1 oral dose of metronidazole (500 mg) twice daily for 7 days, plus probiotics capsules (Provided by Chr Hansen, Horsholm, Denmark). (65). | Treated with 1 oral dose of metronidazole (500 mg) twice daily for 7 days, plus placebo capsules. (60). | Lactobacillus rhamnosus GR-1 & Lactobacillus reuteri RC-14. | Capsules, contained > 109 viable cells of each strain per dose, twice daily. | Orally | 30 days. Starting on day 1 of metronidazole treatment. Taken at least 1 h after the antibiotic. | 1 follow-up visit at day 30(30 days in total). |
| 3 | Larsson 2008  Drammen, Norway | | Treated with a 7-day course of daily 2% vaginal clindamycin cream (Pfizer Norway Ltd.), followed by gelatine probiotics capsules EcoVag® (Bifodan A/S, Denmark). (50) | A 7-day course of daily same vaginal clindamycin cream, followed by gelatine placebo capsules. (50) | Lactobacillus gasseri (Lba EB01-DSM 14869) & Lactobacillus rhamnosus (Lbp PB01-DSM 14870). | Capsules contained 108–109 freeze-dried lactobacilli per dose. Once daily. | Vaginally | 10 days. Repeated after each menstruation for 3 cycles (4 times in total). | 6 follow-up visits in 6 months (180 days in total, after the 1st, 2nd, 3rd, 4th,5th and 6th menstruation). |
| 4 | Martinez 2009  São Paulo, Brazil | | First treated with a single dose of tinidazole (2 g) plus 2 oral capsules (Provided by Chr Hansen, Horsholm, Denmark, in gelatin capsules), followed by capsules only. (32) | First treated with a single dose of tinidazole (2 g) plus 2 capsules (cellulose, magnesium stearate), followed by capsules only. (32) | Lactobacillus rhamnosus GR-1 & Lactobacillus reuteri RC-14 | Capsules contained 109 viable cells of each strain per dose. 2 capsules once daily in the morning. | Orally | 28 days, starting on the first day of tinidazole use. | 1 follow-up visit at 4 weeks after the treatment (28 days in total). |
| 5 | Mastromarino 2009  Rome, Italy | | Treated with  Lactobacillus tablet (Florisia; VSL Pharmaceuticals, Inc., Towson, MD, USA). (20) | Treated with placebo. (19) | Lactobacillus brevis (CD2), Lactobacillus salivarius subsp.  salicinius (FV2), and Lactobacillus plantarum (FV9) | Tablets contained 109 viable lactobacilli per dose. Once daily at bedtime | Vaginally | 7 days. Initiated after the enrolment visit or after the end of menstruation (if expected within a 7-day period). | 2 follow-up visits at the day 7 & 21(21 days in total). |
| 6 | Hemmerling 2010  San Francisco, USA | | First treated with a standard antibiotic, 0.75% topical metronidazole (MetroGel) for 5 consecutive days before enrollment, followed by vaginal application of single-use applicators LACTIN-V. (18) | First treated with a standard antibiotic, 0.75% topical metronidazole (MetroGel) for 5 consecutive days before enrollment, followed by vaginal placebos. (6) | Lactobacillus crispatus CTV-05 | Capsules contained 2x109 CFUs/dose. Once daily. | Vaginally | 5 consecutive days, followed by once weekly application over 2 consecutive additional weeks (day 1-5, day 12 & 19). | 2 follow-up visits at day 10 & 28(28 days in total). |
| 7 | Bradshaw 2011/2012  Melbourne, Australia | | Treated with 400 mg oral metronidazole (7 days) twice daily, plus oestrogen-containing vaginal probiotics. Manufactured by Medinova's, Switzerland. (150) | Positive control: Treated with same metronidazole plus placebo (12 days). (150)  Negative control:  Treated with same metronidazole/vaginal clindamycin (1 g 2% nocte, 7 days). (150) | Lactobacillus acidophilus KS400 | Pessaries contained > 107 CFUs of live lactobacilli and 0.03 mg oestriol per dose. Once daily at bedtime. | Vaginally | 12 days. | 5 follow-up visits at 0, 1, 2, 3 & 6 months (180 days in total). |
| 8 | Vujic 2013  Central and northwestern Croatia | | Treated with administration of selected lactobacilli called "Lactogyn" (JGL, Rijeka, Croatia). (466) | Treated with identical-looking placebo. (185) | Lactobacillus rhamnosus GR-1 & Lactobacillus reuteri RC-14 | Capsules, contained > 109 CFU per dose. Twice daily. | Orally | 6 weeks | 2 follow-up visits at 6 & 12 weeks (84 days in total) |
| 9 | Vicariotto 2014  Milan, Italy | | Given the active formulation. (24) | Given the placebo. (11) | Lactobacillus fermentum LF15 (DSM 26955) & Lactobacillus plantarum LP01 (LMG P-21021) | Tablets contained 4×108 live cells per dose. Once daily. | Vaginally | First for 7 consecutive nights, then 1 tablet every 3 days for 3 weeks. Finally, once a week in the following month. | 2 follow-up visits at the days 28, 56(56 days in total). |
| 10 | Heczko 2015  Krakow & Warsaw, Poland | | Given a standard treatment (500 mg oral metronidazole twice daily for 7 days), followed by the active formulation. (285) | Given a standard treatment (500 mg oral metronidazole twice daily for 7 days), followed by the placebo. (293) | Lactobacillus fermentum 57A, Lactobacillus plantarum 57B & Lactobacillus gasseri 57C. | Capsules, contained > 108 CFU per dose. Twice daily. | Orally | First twice daily for 10 days, then check for symptoms at visit II (No signs of the infection: take once daily for 10 days in the peri-menstrual period for the next 3 months. Metronidazole-resistant G.vaginalis infection: Oral clindamycin. AV: susceptible antibiotic+ probiotics/placebo twice daily for 10 days) | 5 follow-up visits at 0, 1, 2, 3 & 4 months. (120 days in total), Visits III-V occurred for all patients within 7 days after completion of each menstrual period). Patients with symptoms at visit II will be asked for visit II bis, and if secondary treatment was successful they were to proceed for the next 3 months. |
