# Supplementary material for: Probiotics for the Treatment of Bacterial Vaginosis: A Meta-Analysis
Source: Int J Environ Res Public Health. 2019 Oct 12;16(20):3859. doi: 10.3390/ijerph16203859 (PMC6848925; doi:10.3390/ijerph16203859)
Supplement: Supplementary file 1 [file ijerph-16-03859-s001.zip › Supplementary files/Supplementary file 4 - standardized form - outcome.docx]

**Supplementary file 4** Standardized form of data extraction - Outcomes

| **No.** | **Basic information** | **Outcomes** | | | | |
| --- | --- | --- | --- | --- | --- | --- |
|  | **Author, publication year, study site, country** | **Adherence** | **Recovery rates (95% CI, intent to treat analysis, time)** | **Adverse events (95% CI, intent to treat analysis, time)** | **Colonization efficiency of lactobacilli (95% CI, intent to treat analysis, time)** | **Nugent score (time)** |
| 1 | Eriksson 2005, Finland, Norway & Sweden | Lost to follow-up: 15/placebo arm, 15/treatment arm (30).  Drop-out: 17/placebo arm, 21/treatment arm (38). | RR=0.89 (0.69-1.16, after the 2nd menstruation) | Candida infection: RD= 0.04 (-0.60-0.49, in 2 menstrual cycles)  Itching & burning: RD= -0.24 (-0.67-0.20, in 2 menstrual cycles) | NA | 3.85(SD=2.9, probiotics, after the 2nd menstruation)  3.47(SD=2.7, placebo, after the 2nd menstruation) |
| 2 | Anukam 2006a, Benin City, Nigeria | Lost to follow-up: 16/placebo arm, 3/treatment arm at the day 30. (19) | RR=1.73 (1.20-2.49, day 30) | Headache: RD= 0.04(-0.02-0.11, in 30 days) | OR=21.15 (4.68-95.50, day 30) | 4.63(SD=2.93, placebo, day 30)  1.9(SD=1.16, probiotics, day 30) |
| 3 | Larsson 2008, Drammen, Norway | Lost to follow-up: 6/placebo arm, 3/treatment arm at the day 30.(9)  Withdrawal: 5/placebo arm, 10/treatment arm due to the failed treatment at the day 30.(10) | RR=0.95 (0.76-1.18, day 28)  RR=1.25 (0.79-1.96, day 180) | In total: RD=0.07(-0.14-0.28, during 30 days)  Candida infection: RD=-5.07/PY (-14.16-4.02, during 30 days) | NA | NA |
| 4 | Martinez 2009, São Paulo, Brazil | Lost to follow-up: None. (0). | RR=1.75 (1.21-2.53, day 28) | Headache: RD= 0.41/PY (-0.39-1.21, during 28 days)  Candida infection: RD=1.22/PY (-0.56-3.00, during 28 days) | NA | NA |
| 5 | Mastromarino 2009, Rome, Italy | Drop out: 3/placebo arm, 2/treatment arm at the day 7 & 21. (1 required antibiotic therapy and 4 did not return for the follow-up visits, excluded in study group).(5) | RR=12.63 (1.87-85.46, day 7)  RR=5.05 (1.32-19.32, day 21) | RD=0.00/PY (During 21 days) | OR=4.00 (1.71-9.35, day 7)  OR=15.17 (2.84-81.09, day 21) | 7.4(SD=1.3, probiotics, baseline)  7.7(SD=0.9, placebo, baseline)  7.6(SD=1.1, placebo, day 7)  2(SD=1.9, probiotics, day 7)  7.4(SD=1.5, placebo, day 21)  4.3(SD=3.3, probiotics, day 21) |
| 6 | Hemmerling 2010, San Francisco, USA | Withdrawal: 1/placebo arm, 2/treatment arm during 28 days due to the adverse events. (2) | RR=1.56(0.67—3.59, day 28) | In total: RD=-23.17/PY (-52.40-6.05, in 28 days)  Genitourinary:  RD=-19.55/PY (-45.46-6.35, in 28 days)  Related: RD=-13.03/PY (-38.98-12.91, in 28 days)  Moderate severity: RD=-3.26/PY (-20.50-13.98, in 28 days) | OR=1.30 (0.18-9.47, day 28) | NA |
| 7 | Bradshaw 2011/2012, Melbourne, Australia | Lost to follow-up: 15/placebo arm, 10/antibiotic arm, 17/ probiotics arm before day 30. (42)  57 new lost before 180. (57) | RR=1.03 (0.96-1.11, during 30 days)  RR=1.03 (0.80-1.33, in total 112.5 PY) | In total: RD (placebo + antibiotics) =-0.11/PY (-0.57-0.35, in total112.5 PY) | OR=0.90 (0.55-1.48, during 30 days)  OR=0.93 (0.60-1.43, during 180 days) | NA |
| 8 | Vujic 2013, Central and northwestern Croatia | Lost to follow-up: 15/placebo arm, 22/probiotics arm. (37)  Poor compliance: 9/placebo arm, 11/probiotics arm. (20). | RR= 2.46(1.85-3.26, day42)  RR= 0.27(0.20-0.34, day84) | NA | OR=7.38 (4.90-11.20) | NA |
| 9 | Vicariotto 2014, Milan, Italy | 1 drop out in placebo group during the 2nd month. | RR= 3.21(0.88-11.75, day28)  RR= 1.83(0.46-7.25, day56) | NA | NA | 8.5(SD=1.1, probiotics, baseline)  7.9(SD=1.0, placebo, baseline)  8.2(SD=1.3, placebo, day 28)  3.5(SD=1.9, probiotics, day 28)  7.7(SD=1.1, placebo, day 56)  4.3(SD=1.80, probiotics, day 56) |
| 10 | Heczko 2015, Krakow & Warsaw, Poland | Lost to follow-up: 23 before the day 30, 41 before the day 180. (64)  Poor compliance: 42 before the day 30, 27 before the day 180. (69)  Drop out for any other reason: 5 before the day 30, 14 before the day 180. (19)  Pregnant: 2 before the day 30, 4 before the day 180. (6)  Monilial infection: 59 before the day 30, 19 before the day 180 (78)  Misdiagnose: 261 before the day 30. (261) | RR=0.91 (0.73-1.14, day 30)  RR=0.96 (0.64-1.42, day 60)  RR=0.86 (0.55-1.35, day 90)  RR=0.94 (0.59-1.48, day 180) | NA | NA | 2.9(SD=2.58, probiotics, day 60)  3.0(SD=2.54, placebo, day 60)  2.4(SD=2.50, placebo, day 90)  2.1(SD=2.01, probiotics, day 90)  1,8(SD=2.52, placebo, day 120)  1.7(SD=2.18, probiotics, day 120) |

BV=bacterial vaginosis, VVC= vulvovaginal candidiasis, NA=not available, IUD=intrauterine device, OR=odd ratio, RR=risk ratio, RD=risk difference, SD=standard deviation.
