# Supplementary material for: Probiotics for the Treatment of Bacterial Vaginosis: A Meta-Analysis
Source: Int J Environ Res Public Health. 2019 Oct 12;16(20):3859. doi: 10.3390/ijerph16203859 (PMC6848925; doi:10.3390/ijerph16203859)

Original Studies: Efficacy after a normal menstrual cycle (around the 30th day after intervention).

Study or Subgroup	Probiotics Events	Probiotics Total	Control Events	Control Total	Weight	Risk Ratio M-H, Random, 95% CI	Year
**1.6.1 APT: White-dominant (Caucasians >50%)**							
Eriksson 2005	51	106	60	111	12.6%	0.89 [0.69, 1.16]	2005
Larsson 2008	37	50	39	50	13.0%	0.95 [0.76, 1.18]	2008
Bradshaw 2011	104	133	102	135	13.8%	1.03 [0.91, 1.18]	2011
Heczko 2015	96	285	108	293	13.0%	0.91 [0.73, 1.14]	2015
**Subtotal (95% CI)**		**574**		**589**	**52.5%**	**0.98 [0.89, 1.07]**	
Total events	288		309				
Heterogeneity: Tau² = 0.00; Chi² = 1.90, df = 3 (P = 0.59); I² = 0%							
Test for overall effect: Z = 0.48 (P = 0.63)							
**1.6.2 APT: Multi-ethnic (Caucasians ≤ 50%)**							
Anukam 2006	43	65	23	60	11.3%	1.73 [1.20, 2.49]	2006
Martinez 2009	28	32	16	32	11.3%	1.75 [1.21, 2.53]	2009
Hemmerling 2010	14	18	3	6	6.1%	1.56 [0.67, 3.59]	2010
**Subtotal (95% CI)**		**115**		**98**	**28.7%**	**1.72 [1.34, 2.21]**	
Total events	85		42				
Heterogeneity: Tau² = 0.00; Chi² = 0.06, df = 2 (P = 0.97); I² = 0%							
Test for overall effect: Z = 4.28 (P < 0.0001)							
**1.6.3 POT**							
Mastromarino 2009	12	20	2	19	3.1%	5.70 [1.46, 22.18]	2009
Vujic 2013	243	428	40	173	12.3%	2.46 [1.85, 3.26]	2013
Vicariotto 2014	14	24	2	11	3.3%	3.21 [0.88, 11.75]	2014
**Subtotal (95% CI)**		**472**		**203**	**18.8%**	**2.57 [1.96, 3.37]**	
Total events	269		44				
Heterogeneity: Tau² = 0.00; Chi² = 1.54, df = 2 (P = 0.46); I² = 0%							
Test for overall effect: Z = 6.80 (P < 0.00001)							
**Total (95% CI)**		**1161**		**890**	**100.0%**	**1.39 [1.06, 1.82]**	
Total events	642		395				
Heterogeneity: Tau² = 0.13; Chi² = 66.02, df = 9 (P < 0.00001); I² = 86%							
Test for overall effect: Z = 2.36 (P = 0.02)							
Test for subgroup differences: Chi² = 55.27, df = 2 (P < 0.00001), I² = 96.4%							

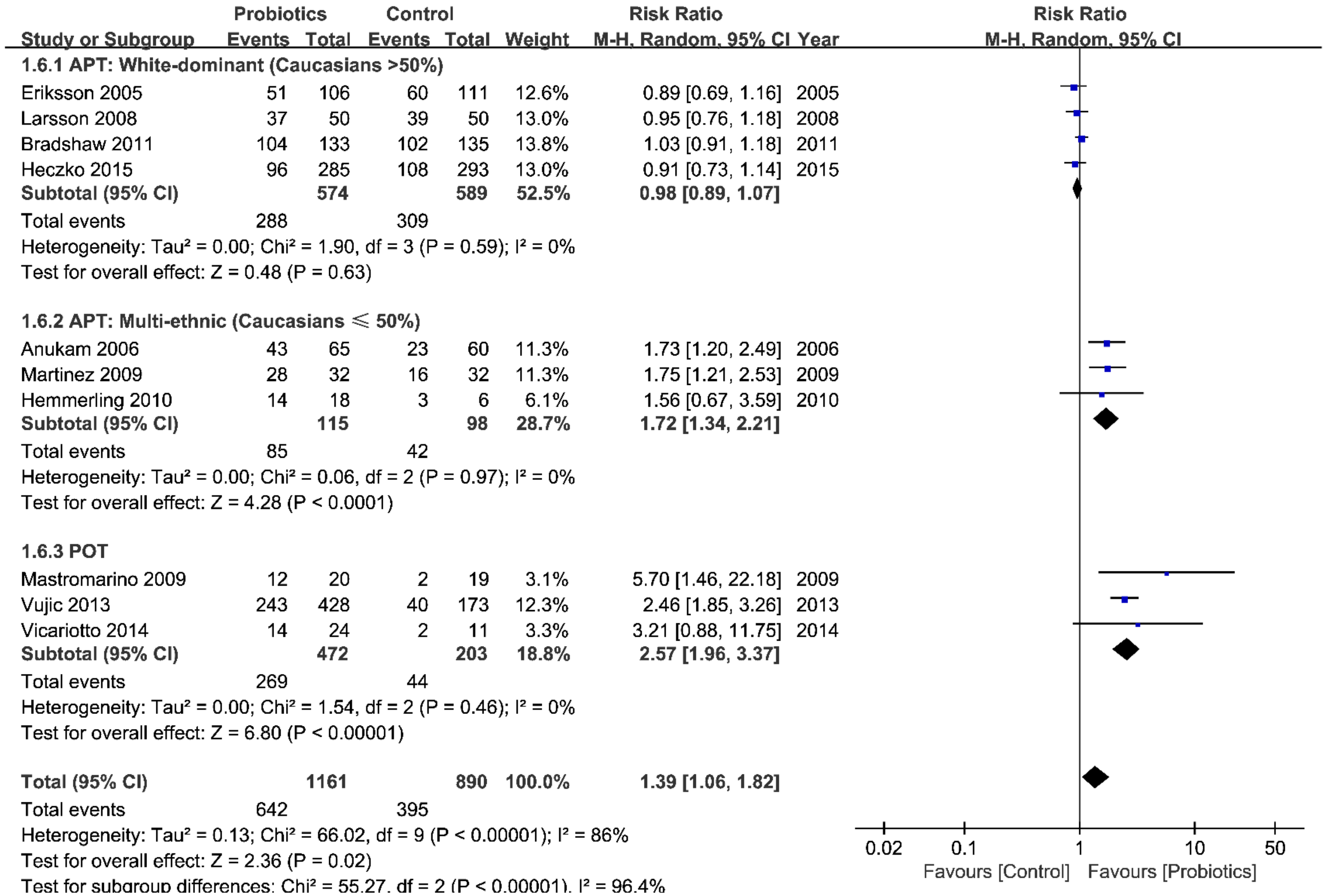

Add studies with abstract only, around Day 30

Study or Subgroup	Experimental Events	Experimental Total	Control Events	Control Total	Weight	Risk Ratio M-H, Random, 95% CI
**7.1.1 APT: White-dominant (Caucasians >50%)**						
Bradshaw 2011	104	133	102	135	13.4%	1.03 [0.91, 1.18]
Eriksson 2005	51	106	60	111	12.2%	0.89 [0.69, 1.16]
Heczko 2015	96	285	108	293	12.6%	0.91 [0.73, 1.14]
Larsson 2008	37	50	39	50	12.6%	0.95 [0.76, 1.18]
**Subtotal (95% CI)**		**574**		**589**	**50.9%**	**0.98 [0.89, 1.07]**
Total events	288		309			
Heterogeneity: Tau² = 0.00; Chi² = 1.90, df = 3 (P = 0.59); I² = 0%						
Test for overall effect: Z = 0.48 (P = 0.63)						
**7.1.2 APT: Multi-ethnic (Caucasians ⩽ 50%)**						
Anukam 2006	43	65	23	60	11.0%	1.73 [1.20, 2.49]
Hemmerling 2010	14	18	3	6	5.9%	1.56 [0.67, 3.59]
Martinez 2009	28	32	16	32	11.0%	1.75 [1.21, 2.53]
**Subtotal (95% CI)**		**115**		**98**	**27.9%**	**1.72 [1.34, 2.21]**
Total events	85		42			
Heterogeneity: Tau² = 0.00; Chi² = 0.06, df = 2 (P = 0.97); I² = 0%						
Test for overall effect: Z = 4.28 (P < 0.0001)						
**7.1.3 POT**						
Mastromarino 2009	12	20	2	19	3.1%	5.70 [1.46, 22.18]
Parent 1996	7	17	2	15	2.9%	3.09 [0.75, 12.65]
Vicariotto 2014	14	24	2	11	3.3%	3.21 [0.88, 11.75]
Vujic 2013	243	428	40	173	12.0%	2.46 [1.85, 3.26]
**Subtotal (95% CI)**		**489**		**218**	**21.2%**	**2.59 [1.98, 3.38]**
Total events	276		46			
Heterogeneity: Tau² = 0.00; Chi² = 1.60, df = 3 (P = 0.66); I² = 0%						
Test for overall effect: Z = 6.98 (P < 0.00001)						
**Total (95% CI)**		**1178**		**905**	**100.0%**	**1.42 [1.09, 1.86]**
Total events	649		397			
Heterogeneity: Tau² = 0.14; Chi² = 68.44, df = 10 (P < 0.00001); I² = 85%						
Test for overall effect: Z = 2.56 (P = 0.01)						
Test for subgroup differences: Chi² = 57.11, df = 2 (P < 0.00001), I² = 96.5%						

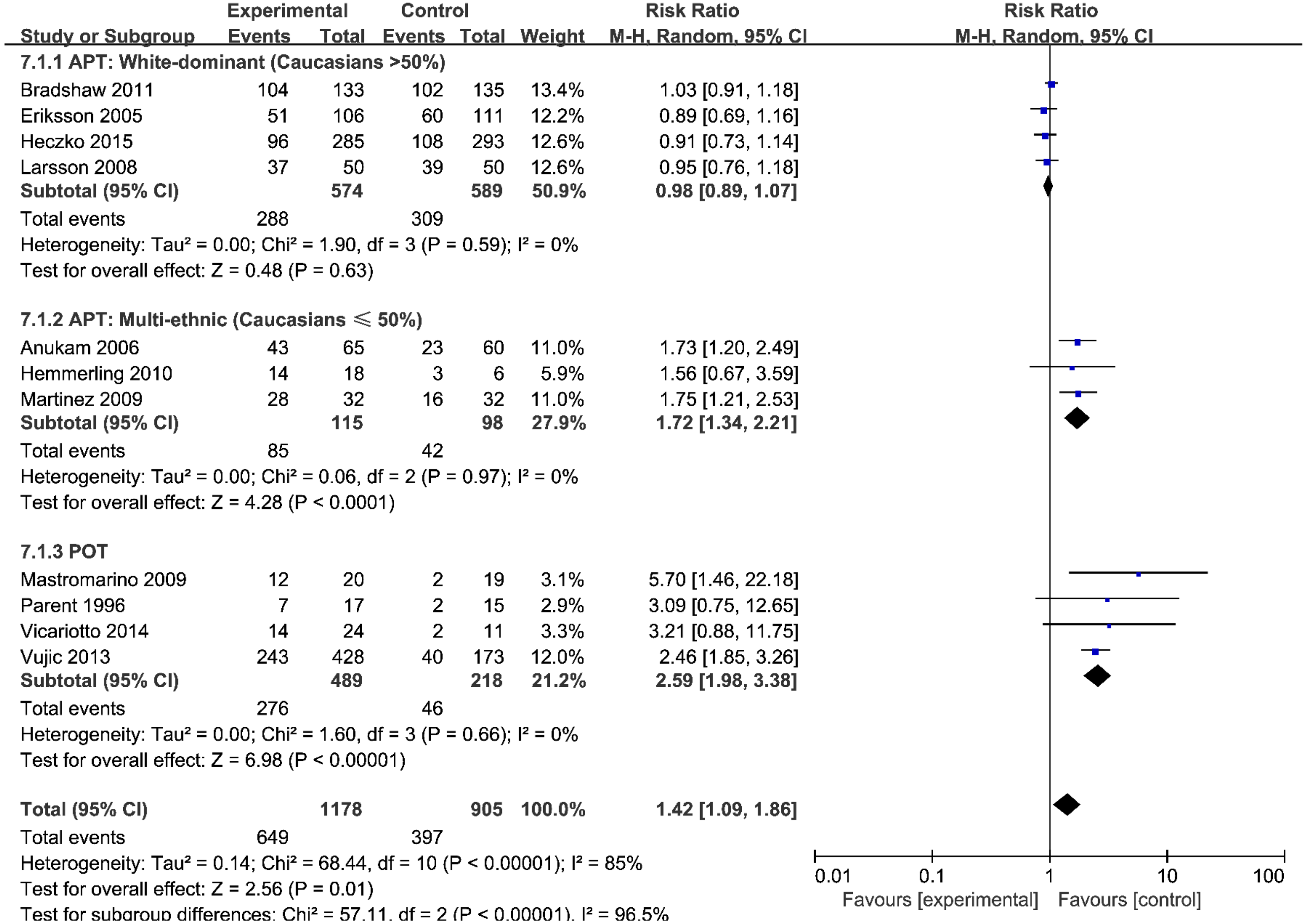

Add studies with food or dietary supplements, around Day 30

Study or Subgroup	Experimental Events	Experimental Total	Control Events	Control Total	Weight	Risk Ratio M-H, Random, 95% CI
**7.4.1 APT: White-dominant (Caucasians >50%)**						
Bradshaw 2011	104	133	102	135	11.8%	1.03 [0.91, 1.18]
Eriksson 2005	51	106	50	111	10.4%	1.07 [0.80, 1.42]
Heczko 2015	96	285	108	293	11.1%	0.91 [0.73, 1.14]
Larsson 2008	37	50	39	50	11.1%	0.95 [0.76, 1.18]
**Subtotal (95% CI)**		**574**		**589**	**44.4%**	**1.00 [0.91, 1.10]**
Total events	288		299			
Heterogeneity: $Tau^2 = 0.00$; $Chi^2 = 1.40$, df = 3 (P = 0.71); $I^2 = 0\%$						
Test for overall effect: Z = 0.02 (P = 0.98)						
**7.4.2 APT: Multi-ethnic (Caucasians ≤ 50%)**						
Anukam 2006	43	65	23	60	9.5%	1.73 [1.20, 2.49]
Hemmerling 2010	14	18	3	6	4.9%	1.56 [0.67, 3.59]
Martinez 2009	28	32	16	32	9.4%	1.75 [1.21, 2.53]
**Subtotal (95% CI)**		**115**		**98**	**23.8%**	**1.72 [1.34, 2.21]**
Total events	85		42			
Heterogeneity: $Tau^2 = 0.00$; $Chi^2 = 0.06$, df = 2 (P = 0.97); $I^2 = 0\%$						
Test for overall effect: Z = 4.28 (P < 0.0001)						
**7.4.3 POT**						
Laue 2017	17	18	11	18	9.3%	1.55 [1.05, 2.27]
Mastromarino 2009	12	20	2	19	2.4%	5.70 [1.46, 22.18]
Shalve 1996	16	23	9	23	7.1%	1.78 [1.00, 3.17]
Vicariotto 2014	14	24	2	11	2.6%	3.21 [0.88, 11.75]
Vujic 2013	243	428	40	173	10.4%	2.46 [1.85, 3.26]
**Subtotal (95% CI)**		**513**		**244**	**31.9%**	**2.13 [1.50, 3.01]**
Total events	302		64			
Heterogeneity: $Tau^2 = 0.06$; $Chi^2 = 7.52$, df = 4 (P = 0.11); $I^2 = 47\%$						
Test for overall effect: Z = 4.27 (P < 0.0001)						
**Total (95% CI)**		**1202**		**931**	**100.0%**	**1.45 [1.14, 1.83]**
Total events	675		405			
Heterogeneity: $Tau^2 = 0.12$; $Chi^2 = 66.16$, df = 11 (P < 0.00001); $I^2 = 83\%$						
Test for overall effect: Z = 3.05 (P = 0.002)						
Test for subgroup differences: $Chi^2 = 30.25$, df = 2 (P < 0.00001), $I^2 = 93.4\%$						

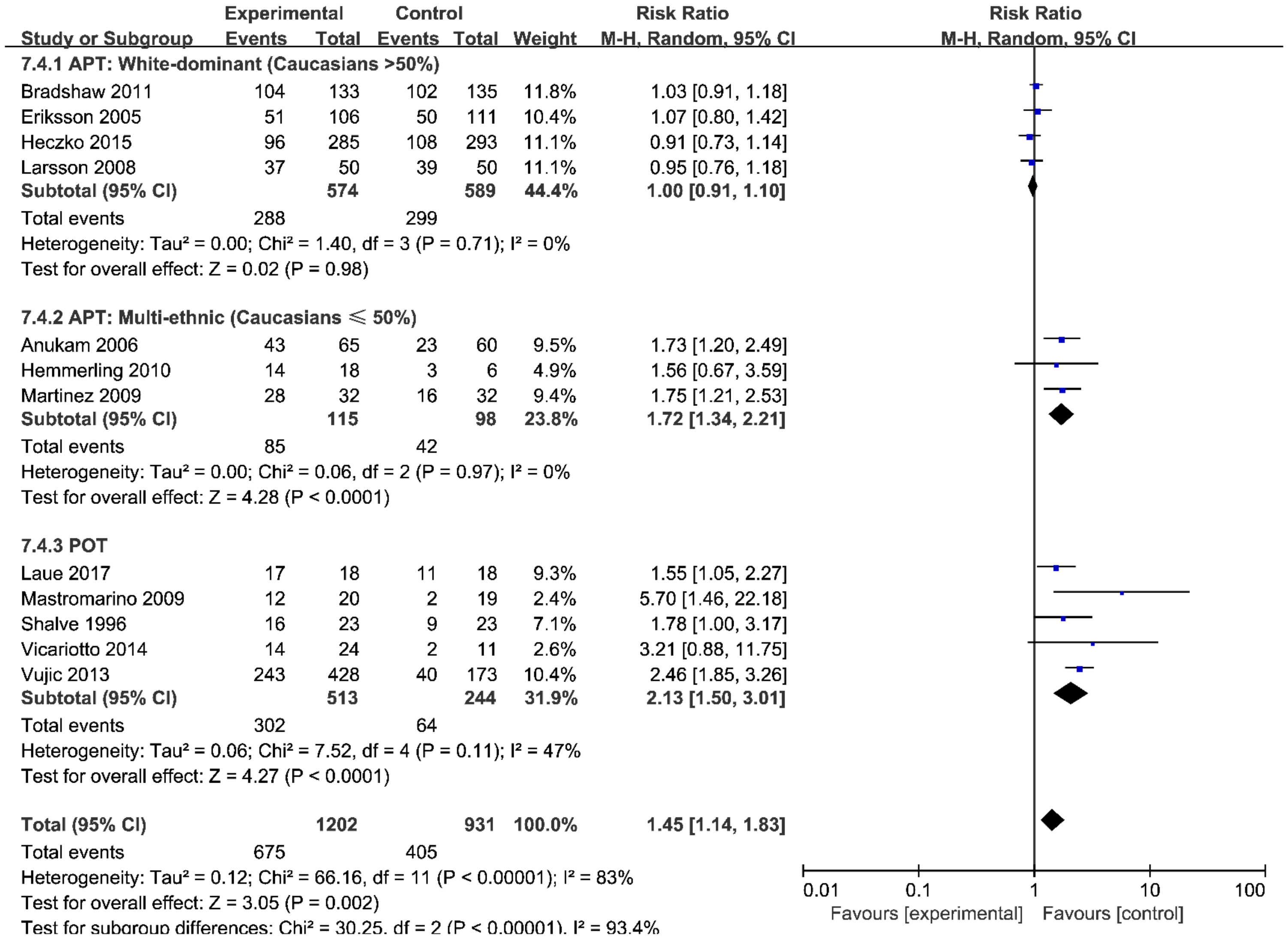

Add studies with high risk of bias, around Day 30

Study or Subgroup	Experimental Events	Experimental Total	Control Events	Control Total	Weight	Risk Ratio M-H, Random, 95% CI
**7.2.1 APT: White-dominant (Caucasians >50%)**						
Bradshaw 2011	104	133	102	135	11.5%	1.03 [0.91, 1.18]
Eriksson 2005	51	106	50	111	9.8%	1.07 [0.80, 1.42]
Heczko 2015	96	285	108	293	10.6%	0.91 [0.73, 1.14]
Larsson 2008	37	50	39	50	10.6%	0.95 [0.76, 1.18]
Marcone 2008 (High risk)	37	42	34	42	11.0%	1.09 [0.91, 1.31]
Marcone 2010 (High risk)	22	25	21	25	10.6%	1.05 [0.84, 1.31]
**Subtotal (95% CI)**		**641**		**656**	**64.1%**	**1.02 [0.94, 1.10]**
Total events	347		354			
Heterogeneity: Tau² = 0.00; Chi² = 2.32, df = 5 (P = 0.80); I² = 0%						
Test for overall effect: Z = 0.51 (P = 0.61)						
**7.2.2 APT: Multi-ethnic (Caucasians ⩽ 50%)**						
Anukam 2006	43	65	23	60	8.8%	1.73 [1.20, 2.49]
Hemmerling 2010	14	18	3	6	4.1%	1.56 [0.67, 3.59]
Martinez 2009	28	32	16	32	8.7%	1.75 [1.21, 2.53]
**Subtotal (95% CI)**		**115**		**98**	**21.5%**	**1.72 [1.34, 2.21]**
Total events	85		42			
Heterogeneity: Tau² = 0.00; Chi² = 0.06, df = 2 (P = 0.97); I² = 0%						
Test for overall effect: Z = 4.28 (P < 0.0001)						
**7.2.3 POT**						
Hallen 1992 (High risk)	3	30	0	30	0.5%	7.00 [0.38, 129.93]
Mastromarino 2009	12	20	2	19	1.9%	5.70 [1.46, 22.18]
Vicariotto 2014	14	24	2	11	2.1%	3.21 [0.88, 11.75]
Vujic 2013	243	428	40	173	9.8%	2.46 [1.85, 3.26]
**Subtotal (95% CI)**		**502**		**233**	**14.4%**	**2.59 [1.98, 3.40]**
Total events	272		44			
Heterogeneity: Tau² = 0.00; Chi² = 1.99, df = 3 (P = 0.57); I² = 0%						
Test for overall effect: Z = 6.90 (P < 0.00001)						
**Total (95% CI)**		**1258**		**987**	**100.0%**	**1.32 [1.08, 1.63]**
Total events	704		440			
Heterogeneity: Tau² = 0.09; Chi² = 68.76, df = 12 (P < 0.00001); I² = 83%						
Test for overall effect: Z = 2.65 (P = 0.008)						
Test for subgroup differences: Chi² = 53.47, df = 2 (P < 0.00001), I² = 96.3%						

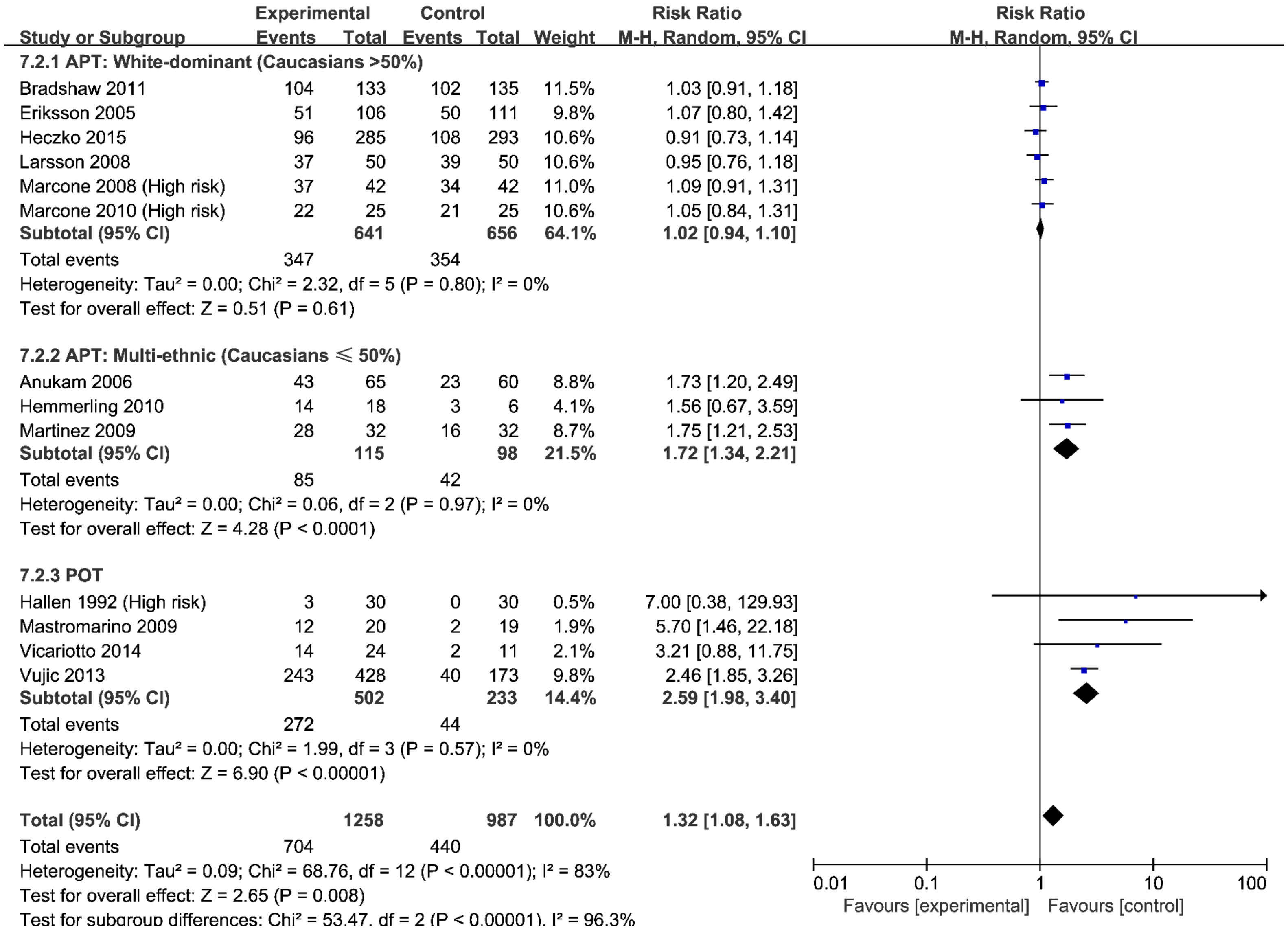

Add unblinded trials, around Day 30

Study or Subgroup	Experimental Events	Experimental Total	Control Events	Control Total	Weight	Risk Ratio M-H, Random, 95% CI
**1.11.1 APT: White-dominant (Caucasians >50%)n**						
Bradshaw 2011	124	133	122	135	11.0%	1.03 [0.96, 1.11]
Heczko 2015	96	285	108	293	10.3%	0.91 [0.73, 1.14]
Larsson 2008	37	50	39	50	10.3%	0.95 [0.76, 1.18]
Marcone 2008 (unblinded)	37	42	34	42	10.5%	1.09 [0.91, 1.31]
Marcone 2010 (unblinded)	21	23	17	23	9.9%	1.24 [0.94, 1.62]
Petricevic 2008 (unblinded)	69	95	31	95	9.5%	2.23 [1.63, 3.05]
**Subtotal (95% CI)**		**628**		**638**	**61.5%**	**1.14 [0.95, 1.38]**
Total events	384		351			
Heterogeneity: $Tau^2 = 0.04$; $Chi^2 = 28.21$, df = 5 ($P < 0.0001$); $I^2 = 82\%$						
Test for overall effect: $Z = 1.45$ ($P = 0.15$)						
**1.11.2 APT: Multi-ethnic (Caucasians ⩽ 50%)**						
Anukam 2006	43	65	23	60	9.1%	1.73 [1.20, 2.49]
Hemmerling 2010	14	18	3	6	5.1%	1.56 [0.67, 3.59]
Martinez 2009	28	32	16	32	9.0%	1.75 [1.21, 2.53]
**Subtotal (95% CI)**		**115**		**98**	**23.1%**	**1.72 [1.34, 2.21]**
Total events	85		42			
Heterogeneity: $Tau^2 = 0.00$; $Chi^2 = 0.06$, df = 2 ($P = 0.97$); $I^2 = 0\%$						
Test for overall effect: $Z = 4.28$ ($P < 0.0001$)						
**1.11.3 POT**						
Mastromarino 2009	12	19	2	16	2.7%	5.05 [1.32, 19.32]
Vicariotto 2014	14	24	2	11	2.9%	3.21 [0.88, 11.75]
Vujic 2013	243	428	40	173	9.8%	2.46 [1.85, 3.26]
**Subtotal (95% CI)**		**471**		**200**	**15.4%**	**2.56 [1.95, 3.36]**
Total events	269		44			
Heterogeneity: $Tau^2 = 0.00$; $Chi^2 = 1.19$, df = 2 ($P = 0.55$); $I^2 = 0\%$						
Test for overall effect: $Z = 6.78$ ($P < 0.00001$)						
**Total (95% CI)**		**1214**		**936**	**100.0%**	**1.47 [1.14, 1.89]**
Total events	738		437			
Heterogeneity: $Tau^2 = 0.15$; $Chi^2 = 123.53$, df = 11 ($P < 0.00001$); $I^2 = 91\%$						
Test for overall effect: $Z = 2.94$ ($P = 0.003$)						
Test for subgroup differences: $Chi^2 = 24.24$, df = 2 ($P < 0.00001$), $I^2 = 91.7\%$						

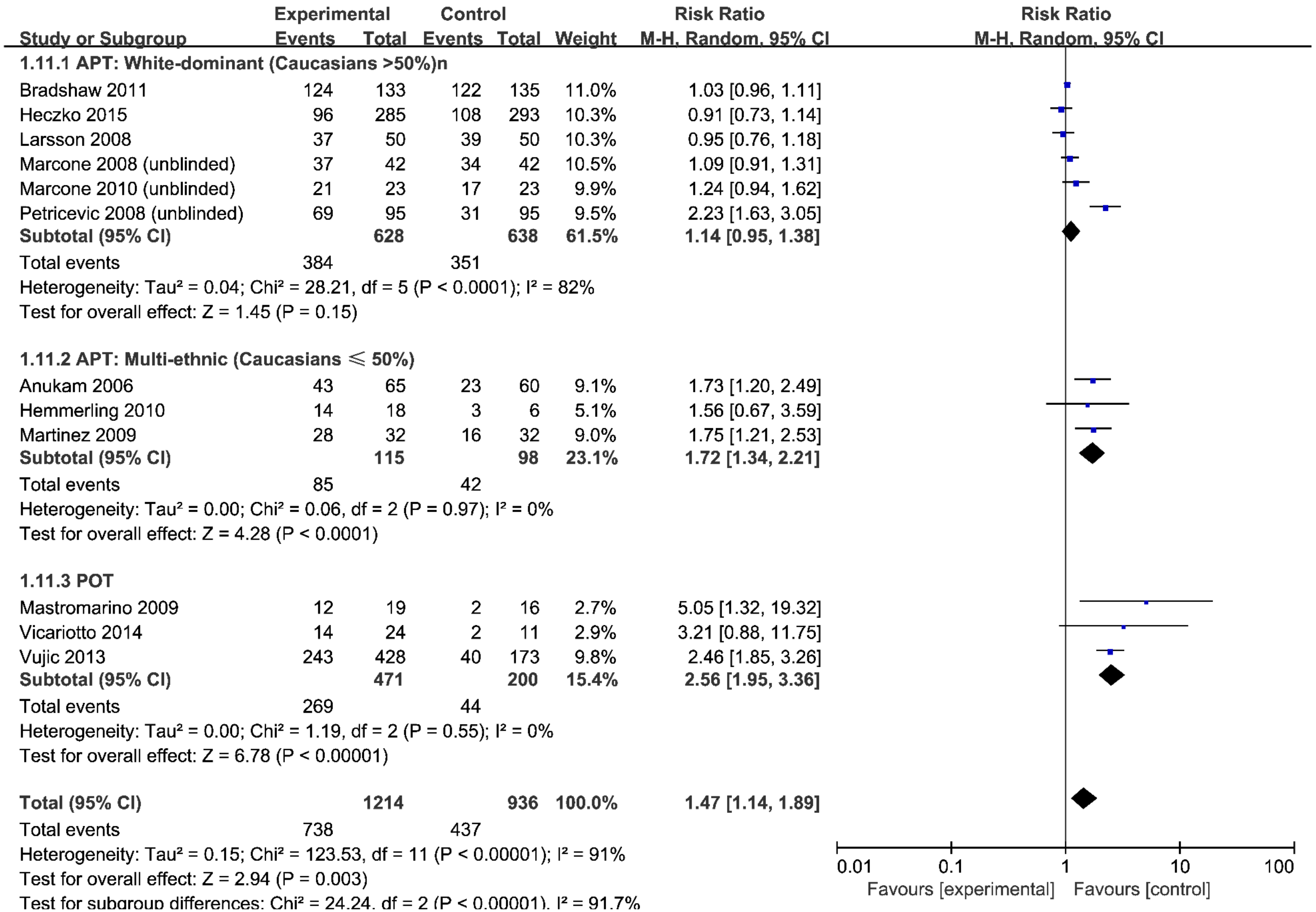

Add studies with high risk of bias, around Day 60

	Experimental		Control			Risk Ratio
Study or Subgroup	Events	Total	Events	Total	Weight	M-H, Fixed, 95% CI
**7.3.1 APT: White-dominant (Caucasians >50%)**						
Bradshaw 2011	63	133	62	135	14.6%	1.03 [0.80, 1.33]
Eriksson 2005	63	112	74	113	17.5%	0.86 [0.70, 1.06]
Heczko 2015	40	285	43	293	10.0%	0.96 [0.64, 1.42]
Larsson 2008	24	47	18	44	4.4%	1.25 [0.79, 1.96]
Marcone 2008 (High risk)	37	42	30	42	7.1%	1.23 [0.99, 1.54]
Marcone 2010 (High risk)	22	25	19	25	4.5%	1.16 [0.89, 1.51]
Recine 2016 (High risk)	113	125	99	125	23.5%	1.14 [1.03, 1.27]
**Subtotal (95% CI)**		**769**		**777**	**81.6%**	**1.05 [0.96, 1.15]**
Total events	362		345			
Heterogeneity: Chi² = 9.02, df = 6 (P = 0.17); I² = 33%						
Test for overall effect: Z = 1.11 (P = 0.26)						
**7.3.2 POT**						
Vicariotto 2014	8	24	1	11	0.3%	3.67 [0.52, 25.84]
Vujic 2013	202	444	52	160	18.1%	1.40 [1.10, 1.79]
**Subtotal (95% CI)**		**468**		**171**	**18.4%**	**1.44 [1.13, 1.84]**
Total events	210		53			
Heterogeneity: Chi² = 0.93, df = 1 (P = 0.33); I² = 0%						
Test for overall effect: Z = 2.93 (P = 0.003)						
**Total (95% CI)**		**1237**		**948**	**100.0%**	**1.12 [1.03, 1.23]**
Total events	572		398			
Heterogeneity: Chi² = 12.79, df = 8 (P = 0.12); I² = 37%						
Test for overall effect: Z = 2.61 (P = 0.009)						
Test for subgroup differences: Chi² = 5.54, df = 1 (P = 0.02), I² = 81.9%						

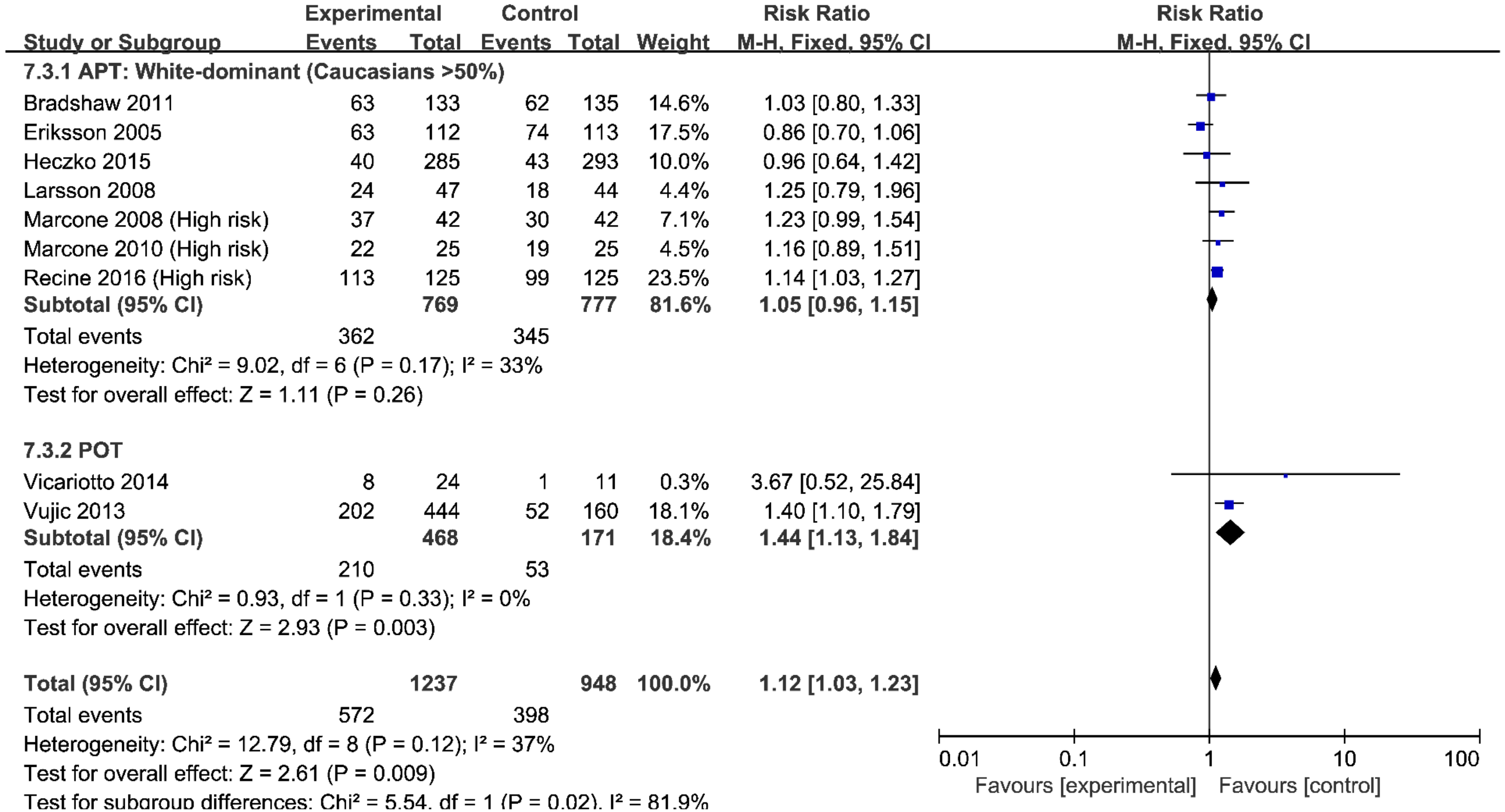

Add studies less than 20 participants, around Day 60

Study or Subgroup	Experimental Events	Experimental Total	Control Events	Control Total	Weight	Risk Ratio M-H, Fixed, 95% CI
**7.5.1 APT: White-dominant (Caucasians >50%)**						
Bradshaw 2011	63	133	62	135	22.0%	1.03 [0.80, 1.33]
Eriksson 2005	63	112	74	113	26.4%	0.86 [0.70, 1.06]
Heczko 2015	40	285	43	293	15.2%	0.96 [0.64, 1.42]
Larsson 2008	24	47	18	44	6.7%	1.25 [0.79, 1.96]
**Subtotal (95% CI)**		**577**		**585**	**70.2%**	**0.97 [0.84, 1.13]**
Total events	190		197			
Heterogeneity: Chi² = 2.70, df = 3 (P = 0.44); I² = 0%						
Test for overall effect: Z = 0.39 (P = 0.70)						
**7.5.3 POT**						
Gille 2012	1	5	8	10	1.9%	0.25 [0.04, 1.48]
Vicariotto 2014	8	24	1	11	0.5%	3.67 [0.52, 25.84]
Vujic 2013	202	444	52	160	27.4%	1.40 [1.10, 1.79]
**Subtotal (95% CI)**		**473**		**181**	**29.8%**	**1.36 [1.07, 1.73]**
Total events	211		61			
Heterogeneity: Chi² = 4.52, df = 2 (P = 0.10); I² = 56%						
Test for overall effect: Z = 2.54 (P = 0.01)						
**Total (95% CI)**		**1050**		**766**	**100.0%**	**1.09 [0.96, 1.23]**
Total events	401		258			
Heterogeneity: Chi² = 13.89, df = 6 (P = 0.03); I² = 57%						
Test for overall effect: Z = 1.30 (P = 0.19)						
Test for subgroup differences: Chi² = 5.57, df = 1 (P = 0.02), I² = 82.0%						

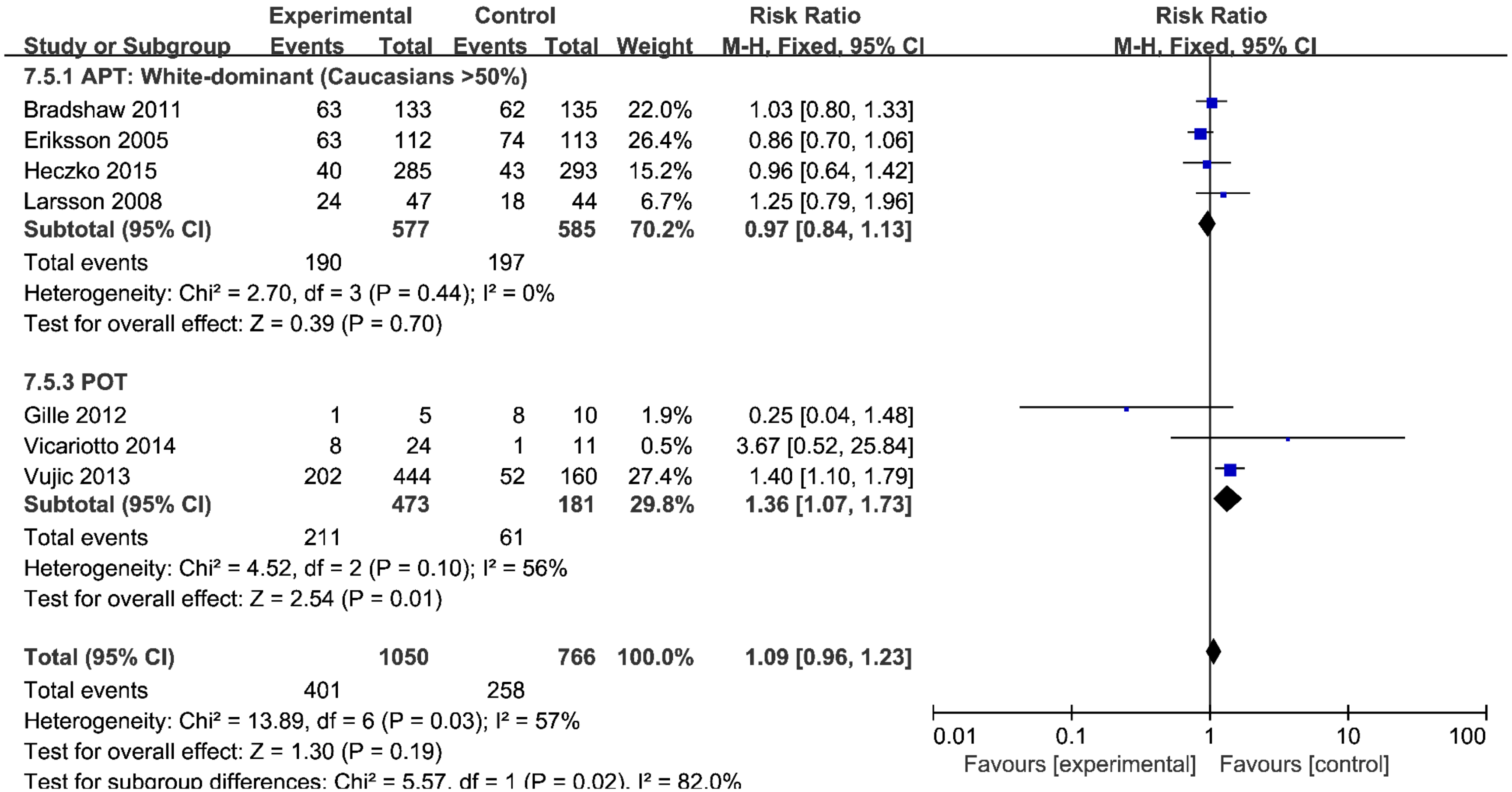

Supplement: Supplementary file 1 [file ijerph-16-03859-s001.zip › Supplementary files/Supplementary file 5 sensitivity analysis.pdf]
