# Supplementary material for: Probiotics for the Treatment of Bacterial Vaginosis: A Meta-Analysis
Source: Int J Environ Res Public Health. 2019 Oct 12;16(20):3859. doi: 10.3390/ijerph16203859 (PMC6848925; doi:10.3390/ijerph16203859)
Supplement: Supplementary file 1 [file ijerph-16-03859-s001.zip › Supplementary files/Table S1 - (simplified).docx]

**Table S1 Characteristics of 10 eligible studies**

| No. | **Basic information**  Author, publication year,  study site, country | **Study design** | **Participants**  N, age range (mean/ median),  Pregnancy status, Ethnic group,  Diagnostic standards of BV | **Intervention**  Type and route,  Follow-up time | **Comparison**  Type and route | **Outcomes** |
| --- | --- | --- | --- | --- | --- | --- |
|  |  |  |  | **APT** |  |  |
| 1 | Eriksson 2005  Finland, Norway & Sweden | Multicentre randomized, double blind, placebo-controlled trial | 217, >18 years (32),  Non-pregnant, Caucasian 178, Other 9, Lost or excluded 68  Amsel's & Nugent's criteria | Clindamycin (ovules vaginally) + probiotics (tampon vaginally),  2 follow-up visits in 2 menstrual cycles (≈60 days in total) | Clindamycin (ovules vaginally) + placebo (tampon vaginally) | Recovery rates, Adverse events |
| 2 | Anukam 2006a  Benin City, Nigeria | Randomized, double blind, placebo-controlled trial | 125, 18-44 years (NA),  Non-pregnant, Black,  Nugent's criteria & BV Blue test | Metronidazole(orally)+ probiotics (capsule orally),  1 follow-up visit at day 30 (30 days in total) | Metronidazole(orally) + placebo (capsule orally) | Recovery rates, Adverse events, Colonization efficiency of lactobacilli |
| 3 | Larsson 2008  Drammen, Norway | Randomized, double-blind, placebo-controlled trial | 100, 18.8-53.6 years (34.3),  Non-pregnant, NA,  Amsel’s criteria | Clindamycin (cream vaginally) + probiotics (capsule vaginally),  6 follow-up visits in 6 months (180 days in total). | Clindamycin (cream vaginally) + placebo (capsule vaginally) | Recovery rates, Adverse events |
| 4 | Martinez 2009  São Paulo, Brazil | Multicentre randomized, double blind, placebo-controlled trial | 64, NA (30.2),  NA, 60% Black/ mulatto, 40% Caucasian,  Amsel's & Nugent's criteria | Tinidazole (capsule orally) + probiotics (capsule orally),  1 follow-up visit at 4 weeks (28 days in total). | Tinidazole (capsules orally) + probiotics (capsule orally) | Recovery rates, Adverse events |
| 5 | Hemmerling 2010  San Francisco, USA | Randomized, double-blind, placebo-controlled trial, phase 2a | 24,18-50 years (29.5),  Non-pregnant, 12 Caucasians, 9 black, 5 others,  Amsel's & Nugent's criteria | Metronidazole (MetroGel) + probiotics (capsule vaginally),  2 follow-up visits at day 10 & 28 (28 days in total). | Metronidazole (MetroGel) +placebo (vaginally) | Recovery rates, Adverse events, Colonization efficiency of lactobacilli |
| 6 | Bradshaw 2011/2012  Melbourne, Australia | Randomized double-blind placebo/antibiotic controlled trial | 268, 18-49 years (27),  Non-pregnant, 312 Australian/British, 136 others, 2 NA,  Amsel's & Nugent's criteria | Metronidazole (orally) + probiotics (pessaries vaginally),  5 follow-up visits at 0, 1, 2, 3 & 6 months (180 days in total). | Positive control: metronidazole (orally) +clindamycin (vaginally)  Negative control:  metronidazole (orally) + placebo (vaginally) | Recurrence rates, Adverse events, Disappearance of lactobacilli |
| 7 | Heczko 2015  Krakow & Warsaw, Poland | Multicentre randomized, double-blind, placebo-controlled trial | 578, 18-50 years (NA),  Non-pregnant, Caucasians,  Asmel's criteria | Metronidazole (orally) + Probiotics (capsule orally),  follow-up visits at 0, 1, 2, 3 & 4 months (120 days in total). | Metronidazole (orally) + Placebo (capsule orally) | Adverse events, Relapse rates, Vaginal pH values, Nugent scores, Counts of vaginal Lactobacillus |
|  |  |  |  | **POT** |  |  |
| 8 | Mastromarino 2009  Rome, Italy | Randomized, double-blind, placebo-controlled trial | 39, 18-NA years (34),  Non-pregnant, NA,  Amsel's & Nugent's criteria | Probiotics (tablet vaginally),  2 follow-up visits at the day 7 & 21 (21 days in total). | Placebo (tablet vaginally) | Recovery rates, Adverse events, Colonization efficiency of lactobacilli, Nugent score |
| 9 | Vujic 2013  Central and northwestern Croatia | Multicentre randomized, double-blind, placebo-controlled trial | 601,18-58 years (33.0),  Non-pregnant, NA,  Nugent's & Asmel's criteria | Probiotics (capsule orally),  2 follow-up visits at 6 & 12 weeks (84 days in total). | Placebo (capsule orally) | Recovery rates, Colonization efficiency of lactobacilli |
| 10 | Vicariotto 2014  Milan, Italy | Randomized, double-blind placebo-controlled trial | 35, 18-50 years (34.7),  Non-pregnant, NA,  Nugent's & Asmel's criteria | Probiotics (tablet orally),  2 follow-up visits at the days 28, 56 (56 days in total). | Placebo (tablet orally) | Recovery rates, Nugent score |

BV= bacterial vaginosis, NA= No data available, APT = antibiotic plus probiotics combination therapy, POT = probiotics-only therapy.
