# Supplementary material for: Probiotics for the Treatment of Bacterial Vaginosis: A Meta-Analysis
Source: Int J Environ Res Public Health. 2019 Oct 12;16(20):3859. doi: 10.3390/ijerph16203859 (PMC6848925; doi:10.3390/ijerph16203859)
Supplement: Supplementary file 1 [file ijerph-16-03859-s001.zip › Supplementary files/Table S2 - bias of each study.docx]

**Table S2 Risk of bias source of 10 studies**

| Bias | Random sequence generation (selection bias) | | Allocation concealment (selection bias) | | Blinding of participants and personnel (performance bias) | | Blinding of outcome assessment (detection bias) | | Incomplete outcome data (attrition bias) | | Selective reporting (reporting bias) | | Other bias | |
| --- | --- | --- | --- | --- | --- | --- | --- | --- | --- | --- | --- | --- | --- | --- |
|  | Authors’ judgement | Support for judgement | Authors’ judgement | Support for judgement | Authors’ judgement | Support for judgement | Authors’ judgement | Support for judgement | Authors’ judgement | Support for judgement | Authors’ judgement | Support for judgement | Authors’ judgement | Support for judgement |
| Eriksson 2005 | unclear | Randomized, no further information | unclear | No information provided | unclear | Double blind, no further information | unclear | Double blind, no further information | low | Missing outcome data balanced in numbers across intervention groups, with similar reasons for missing data across groups | unclear | No information provided | unclear | No information provided |
| Anukam 2006a | low | Randomization was performed using a computer-generated scheme, prepared by the pharmacy. | low | The subjects were allotted numbers, and identical-looking probiotics and placebo capsules were prepared and distributed in numbered containers, again by the pharmacy. | low | Vaginal swab smears were graded by a technician blinded to the subject groups, participants were randomised in a double-blind manner | unclear | No information provided | low | For dichotomous outcome data, the proportion of missing outcomes compared with observed event risk not enough to have a clinically relevant impact on the intervention effect estimate. | unclear | No information provided | unclear | Patients took antibiotics and probiotics at the same time. There's not enough rationale for this patterns. |
| Larsson 2008 | low | The patients were equally allocated 1:1 to either lactobacilli capsules or placebo capsules of identical appearance by block randomisation | unclear | No information provided | low | The placebo vaginal capsules of identical appearance. All study personnel were blind to the treatment throughout the study. | low | All the data were collected and recorded in data sheets before the code of the active or placebo treatment was added. Thereafter no changes in the data sheet were allowed. | low | Missing data have been imputed using the procedure "last observation carried forward". | unclear | No information provided | unclear | The specimen of vaginal fluid was self-swabbed by the patient |
| Martinez 2009 | low | Randomly generated a 5-digit code. (Confirmed by author) | low | Identical vials containing probiotics and placebo were received from the manufacturer in separated boxes with 2 different colors and they were random numbered (a 5-digit code), by staff not participating in the study, in the laboratory at Universida de de Sao Paulo, Brazil. First of all, they prepared small scrips recorded "probiotics/placebo" (half of them with each term). Each scrip was folded a few times to make it opacity. After that they put all those scrips together in a small box, shook it vigorously and took them, one by one, opened them and inserted in the table the word "probiotics/placebo" in front of the 5-digit code previously stated in the table - they followed its ascending numerical order. (Confirmed by author) | low | Probiotics capsule and placebo were identical (white capsules containing the same excipients except the lactobacilli strains in probiotics capsules).(Confirmed by author) | low | The investigators remained blinded to the study codes until all analyses had been completed. | low | No missing outcome data. | low | The study protocol is available but in Portugues.The primary outcome is the efficacy of probiotics capsules(L. rhamnosus GR-1 and L. reuteri RC-14) + single dose of tinidazole (2g) compared to tinidazole + placebo capsules. No secondary outcome was added. (Confirmed by author) | unclear | No information provided |
| Mastromarino 2009 | unclear | No information provided | unclear | No information provided | unclear | Double blind, no further information | unclear | No information provided | high | Potentially inappropriate application of simple imputation. Too many patients lost to follow-up, the proportion of missing outcomes compared with observed event risk not enough to have a clinically relevant impact on the intervention effect estimate. | unclear | No information provided | unclear | No information provided |
| Hemmerling 2010 | unclear | The randomisation scheme was developed by a UCSF pharmacist not otherwise directly involved with study participants. | low | Individual assignments were concealed in sequentially numbered, sealed study drug kits. | low | The active product and the placebo contained the same amount of a gelatin-based preservation matrix containing Food and Drug Administration approved excipients commonly used in dietary products and pharmaceuticals. | low | Participants, investigators, sponsor, and study staff were blinded product allocation. | unclear | The analysis for endpoints was performed within the intent-to-treat (ITT) cohort. | unclear | The study protocol is available but not all of the study's pre-specified (primary and secondary, i.e. cure rate) outcomes that are of interest in the review have been reported in the pre-specified way. | unclear | No information provided |
| Bradshaw 2012 | low | Participants were randomly assigned to one of three study arms in blocks of 15 using a computer-generated sequence. | low | The sequence was produced by a statistician with no clinical input into the trial and securely held by the statistician and nurse packing the vaginal therapies in sealed boxes. At enrolment a research nurse, with no access to the randomisation schedule, gave each woman the next sequentially-numbered sealed box according to the random number sequence. Specific instructions for use of vaginal products were inside the sealed box, which participants were instructed to open at home. | low | Patients were told their vaginal treatment may be an antibiotic, placebo or probiotics, that it may be a cream or pessary, and that the duration of use may be 7 or 12 days, but given no further product details. | low | The participants, the research nurse co-ordinating enrolment and retention of study participants, investigators and microscopists were unaware of the participants' group allocation. | unclear | No information provided | low | The study protocol is available and all of the study's pre-specified (primary and secondary) outcomes that are of interest in the review have been reported in the pre-specified way. | unclear | No information provided |
| Vujic 2013 | low | The randomisation process was done by means of a computer-generated randomisation list. Randomization lists were created using software and methodology described by Saghaei.^1^ | unclear | No information provided | low | Assigning each subject to either "group A" or "group B". Placebo capsules were identical looking. No information for binding of personnel. | high | There were 60 participant excluded during the follow-up period due to not meeting inclusion criteria. But only 27 before the randomisation. | high | "As-treated" analysis done with substantial departure of the intervention received from that assigned at randomisation, but for dichotomous outcome data, the proportion of missing outcomes compared with observed event risk not enough to have a clinically relevant impact on the intervention effect estimate. | unclear | The study protocol is not available, no information provided. | unclear | No information provided |
| Vicariotto 2014 | unclear | No information provided | unclear | No information provided | unclear | Double blind, no further information | unclear | Double blind, no further information | low | Reasons for missing outcome data unlikely to be related to true outcome. | unclear | No information provided | unclear | No information provided |
| Heczko 2015 | low | All eligible women were randomly assigned to one of two study arms (1:1) (using block randomisation with a block size of 12 and equal group ratios) | unclear | No information provided | unclear | Placebo looked identical but contained excipients only. No further information about blinding of personnel. | unclear | Double blind, no further information | unclear | Missing outcome data balanced in numbers across intervention groups, with similar reasons for missing data across groups, but more than 50% data was excluded. | unclear | The study protocol is available but the trial was registered after it was finished(2013). | unclear | No information provided |

1. Saghaei M. Random allocation software for parallel group randomized trials. *BMC Medical Research Methodology.* 2004;4(1):26.
