# Supplementary material for: Probiotics for the Treatment of Bacterial Vaginosis: A Meta-Analysis
Source: Int J Environ Res Public Health. 2019 Oct 12;16(20):3859. doi: 10.3390/ijerph16203859 (PMC6848925; doi:10.3390/ijerph16203859)
Supplement: Supplementary file 1 [file ijerph-16-03859-s001.zip › Supplementary files/Table S3 - safety.docx]

**Table S3 Summary of additional safety analysis results**

| Groups | No. of studies | N total | RD (95% CI) | *P* (overall effect) | *I^2^*, % | *P* (heterogeneity) |
| --- | --- | --- | --- | --- | --- | --- |
| All studies | 10 | 2375 | 0.01 (-0.02 to 0.04) | 0.44 | 0 | 0.78 |
| Gastrointestinal tract | 3 | 947 | 0.02 (-0.02 to 0.07)) | 0.33 | 34 | 0.22 |
| Vaginal itch/soreness | 4 | 1164 | 0.00 (-0.02 to 0.01) | 0.56 | 0 | 0.62 |
| Candida infection | 3 | 321 | 0.00 (-0.05 to 0.05) | 0.92 | 0 | 0.96 |
| Headache | 5 | 1136 | 0.01 ( 0.00 to 0.02) | 0.09 | 0 | 0.76 |
| Preterm labor | 2 | 960 | -0.01 (-0.04 to 0.01) | 0.32 | 0 | 0.98 |
| Others | 10 | 2375 | 0.00 (-0.01 to 0.02) | 0.69 | 0 | 0.58 |

RD = risk differences.
