# Supplementary material for: Probiotics for the Treatment of Bacterial Vaginosis: A Meta-Analysis
Source: Int J Environ Res Public Health. 2019 Oct 12;16(20):3859. doi: 10.3390/ijerph16203859 (PMC6848925; doi:10.3390/ijerph16203859)
Supplement: Supplementary file 1 [file ijerph-16-03859-s001.zip › Supplementary files/Table S4 - sensitivity analysis.docx]

**Table S4 Summary of sensitivity analysis results**

| Groups | No. of studies | N total | RR (95% CI) | *P* (overall effect) | *I^2^*, % | *P* (heterogeneity) |
| --- | --- | --- | --- | --- | --- | --- |
| Clinical cure rate at 30^th^ day |  |  |  |  |  |  |
| Original studies |  |  |  |  |  |  |
| APT: White-dominant | 4 | 1163 | 0.98 (0.89 to 1.07) | 0.63 | 0 | 0.59 |
| APT: Multi-ethnic | 3 | 213 | 1.72 (1.34 to 2.21) | <0.0001 | 0 | 0.97 |
| POT | 4 | 707 | 2.59 (1.98 to 3.38) | <0.00001 | 0 | 0.66 |
| Abstract Only |  |  |  |  |  |  |
| APT: White-dominant | 4 | 1163 | 0.98 (0.89 to 1.07) | 0.63 | 0 | 0.59 |
| APT: Multi-ethnic | 3 | 213 | 1.72 (1.34 to 2.21) | <0.0001 | 0 | 0.97 |
| POT | 4 | 707 | 2.59 (1.98 to 3.38) | <0.00001 | 0 | 0.66 |
| With food or dietary supplements |  |  |  |  |  |  |
| APT: White-dominant | 4 | 1163 | 0.98 (0.89 to 1.07) | 0.63 | 0 | 0.59 |
| APT: Multi-ethnic | 3 | 213 | 1.72 (1.34 to 2.21) | <0.0001 | 0 | 0.97 |
| POT | 5 | 757 | 2.13 (1.50 to 3.01) | <0.0001 | 47 | 0.11 |
| With a high risk of bias |  |  |  |  |  |  |
| APT: White-dominant | 6 | 1297 | 1.02 (0.94 to 1.10) | 0.61 | 0 | 0.80 |
| APT: Multi-ethnic | 3 | 213 | 1.72 (1.34 to 2.21) | <0.0001 | 0 | 0.97 |
| POT | 4 | 735 | 2.59 (1.98 to 3.40) | <0.00001 | 0 | 0.57 |
| Unblinded |  |  |  |  |  |  |
| APT: White-dominant | 6 | 1266 | 1.14 (0.95 to 1.38) | 0.15 | 82 | <0.0001 |
| APT: Multi-ethnic | 3 | 213 | 1.72 (1.34 to 2.21) | <0.0001 | 0 | 0.97 |
| POT | 3 | 675 | 2.57 (1.96 to 3.37) | <0.00001 | 0 | 0.46 |
| Clinical cure rate at 60^th^ day |  |  |  |  |  |  |
| With a high risk of bias |  |  |  |  |  |  |
| APT | 7 | 1546 | 1.05 (0.96 to 1.15) | 0.26 | 33 | 0.17 |
| POT | 2 | 639 | 1.44 (1.13 to 1.84) | 0.003 | 0 | 0.33 |
| less than 20 participants |  |  |  |  |  |  |
| APT | 4 | 1162 | 0.97 (0.84 to 1.13) | 0.70 | 0 | 0.44 |
| POT | 3 | 654 | 1.36 (1.07 to 1.71) | 0.01 | 56 | 0.10 |
| Nugent score at 30^th^ day |  |  |  |  |  |  |
| With food or dietary supplements |  |  |  |  |  |  |
| APT: White-dominant | 3 | 799 | -0.05 (-0.62 to 0.52) | 0.86 | 35 | 0.22 |
| APT: Multi-ethnic | 2 | 73 | -3.83 (-5.10 to -2.57) | <0.00001 | 34 | 0.22 |
| POT | 2 | 170 | -2.70 (-3.42 to -1.99) | <0.00001 | 0 | 0.90 |

RR= risk ratio, APT = antibiotic plus probiotics combination therapy, POT = probiotics-only therapy.
