# Supplementary material for: Probiotics for the Treatment of Bacterial Vaginosis: A Meta-Analysis
Source: Int J Environ Res Public Health. 2019 Oct 12;16(20):3859. doi: 10.3390/ijerph16203859 (PMC6848925; doi:10.3390/ijerph16203859)
Supplement: Supplementary file 1 [file ijerph-16-03859-s001.zip › Supplementary files/Table S5 - attrition.docx]

**Table S5 Attrition analysis of Efficacy on Day 30**

| Method | Numbers of Studies | Sample Size (N) | Pool RR | POT | APT |
| --- | --- | --- | --- | --- | --- |
| Complete case analysis | 10 | 1578 | [1.07, 1.80] | [1.84, 3.12] | [0.89, 1.04] |
| Assuming good outcome | 10 | 2051 | [1.02, 1.59] | [1.40, 1.99] | [0.83, 1.07] |
| Assuming poor outcome | 10 | 2051 | [1.05, 1.85] | [1.96, 3.37] | [0.94, 1.31] |
| Extreme case favouring probiotics | 10 | 2051 | [1.25, 2.38] | [2.40, 4.13] | [0.99, 1.31] |
| Extreme case favouring placebo | 10 | 2051 | [0.84, 1.27] | [1.10, 1.55] | [0.62, 0.94] |

RR= risk ratio, APT = antibiotic plus probiotics combination therapy, POT = probiotics-only therapy.
