# Supplementary figures and images for: Probiotics for the Treatment of Bacterial Vaginosis: A Meta-Analysis
Source: Int J Environ Res Public Health. 2019 Oct 12;16(20):3859. doi: 10.3390/ijerph16203859 (PMC6848925; doi:10.3390/ijerph16203859)

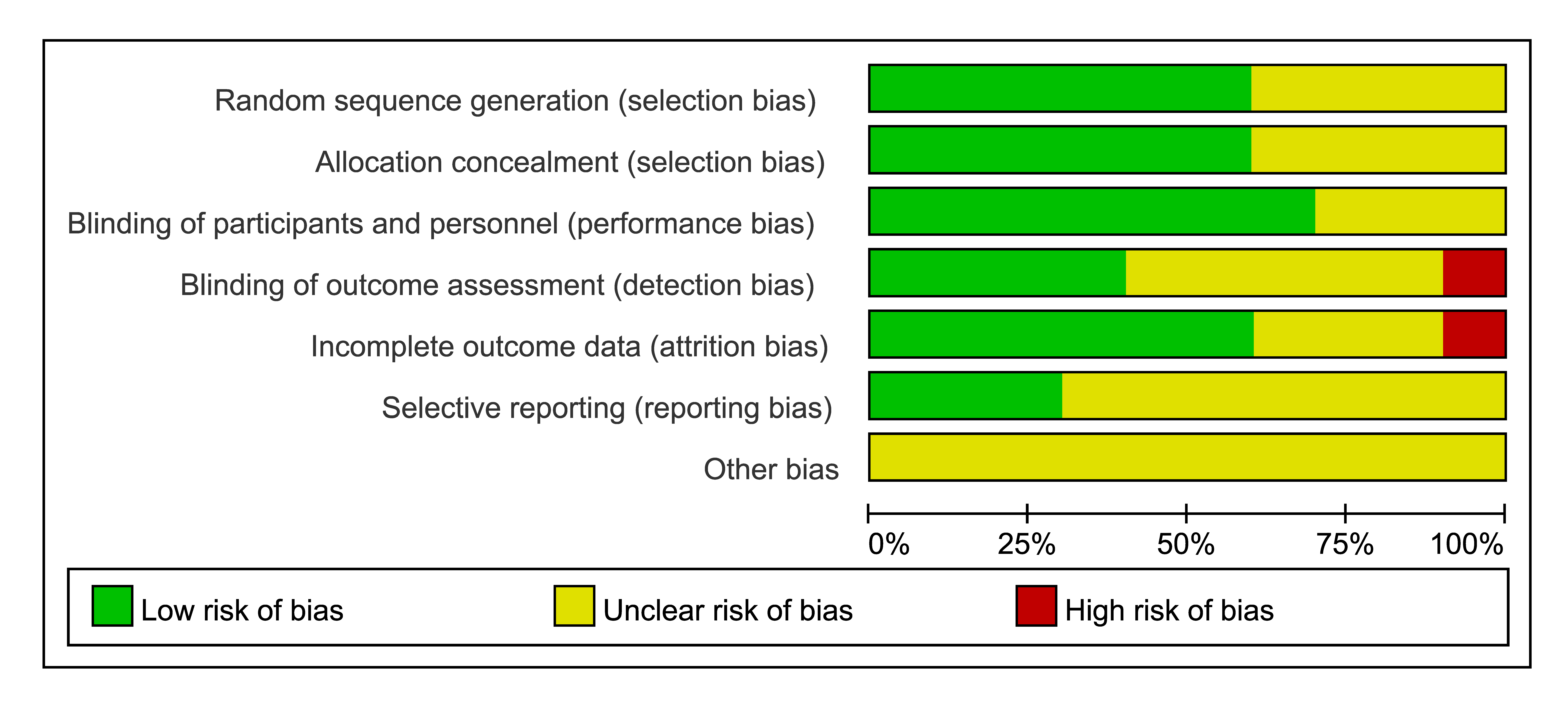

Supplement: Supplementary file 1 [file ijerph-16-03859-s001.zip › Supplementary files/Figure S1 - Risk of bias.tif]

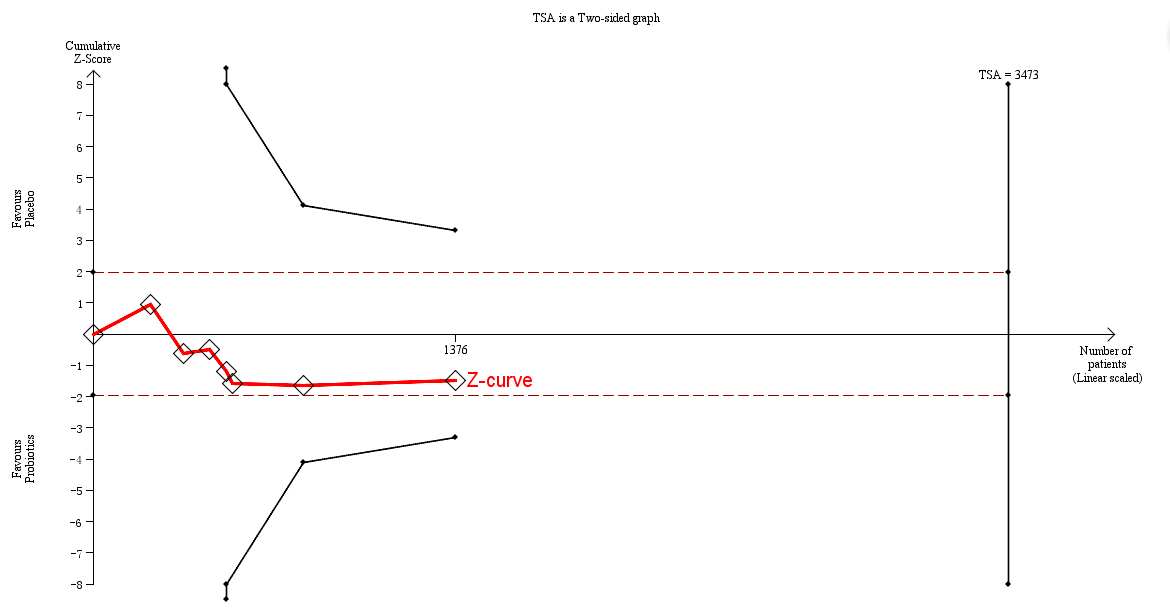

Supplement: Supplementary file 1 [file ijerph-16-03859-s001.zip › Supplementary files/Figure S10.tif]

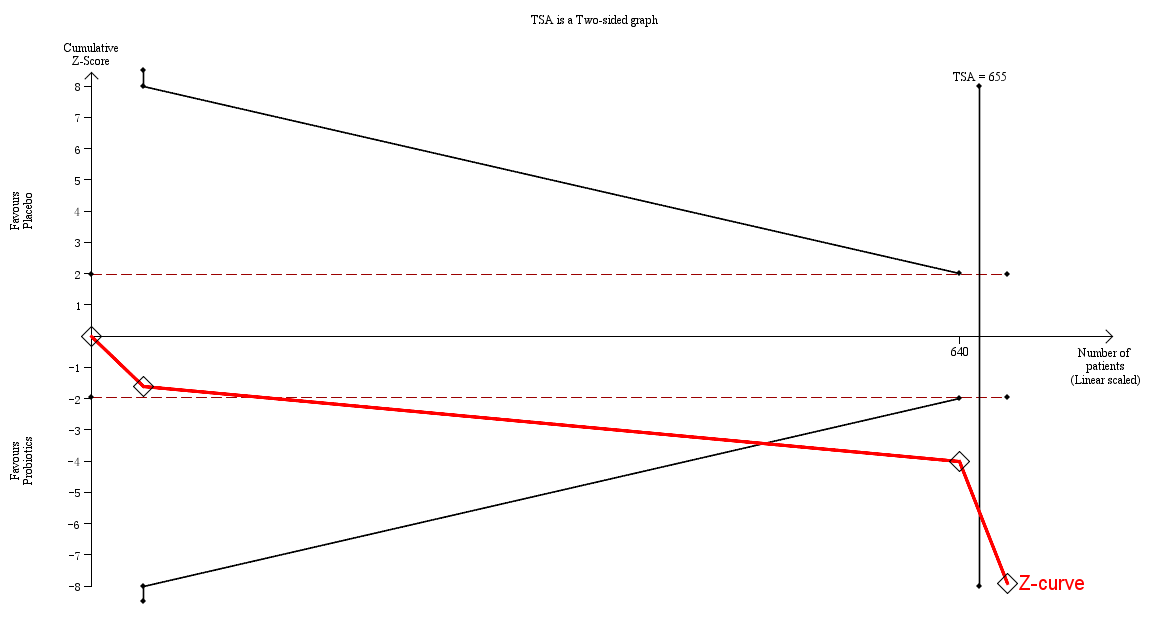

Supplement: Supplementary file 1 [file ijerph-16-03859-s001.zip › Supplementary files/Figure S11.tif]

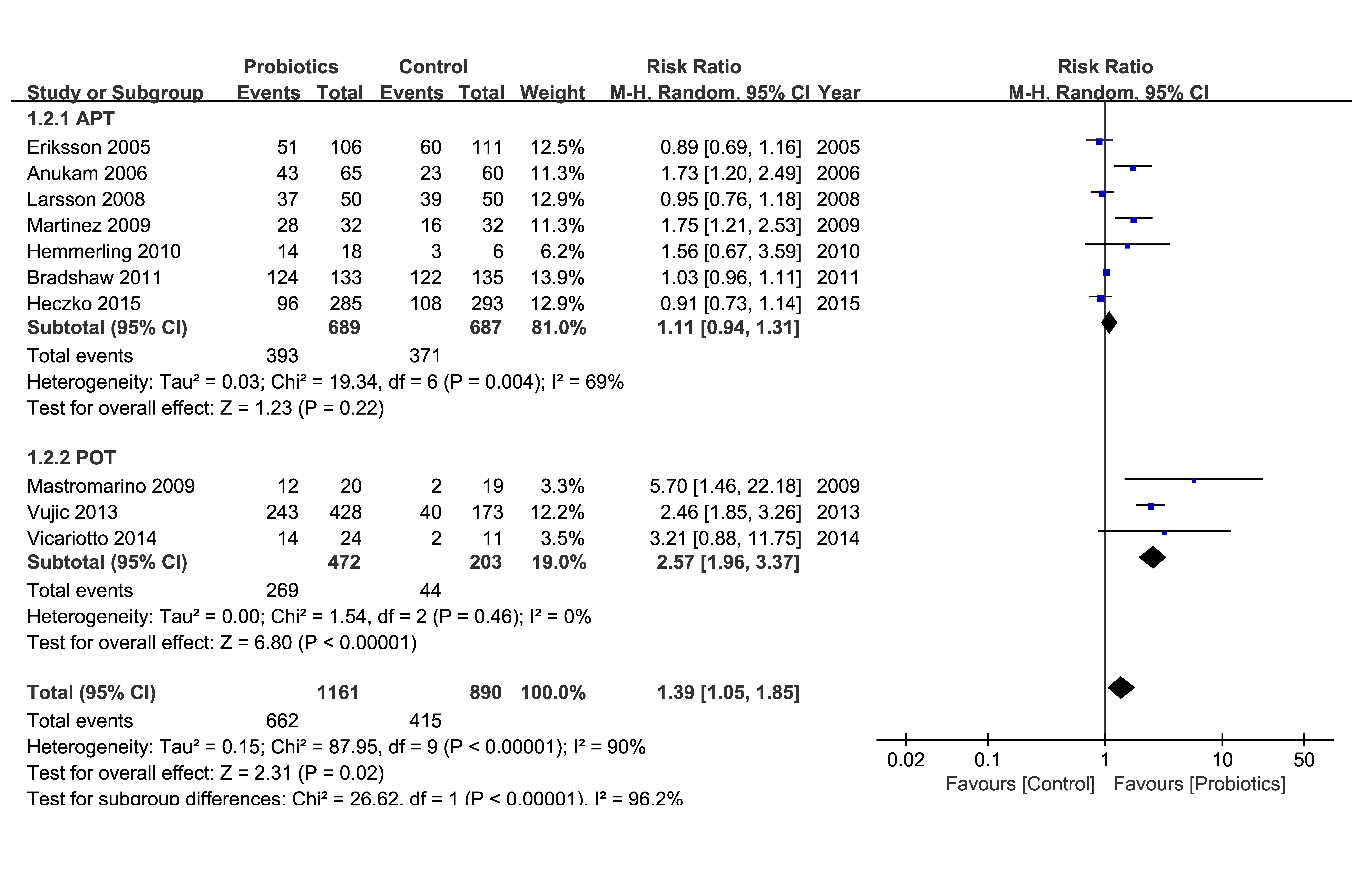

Supplement: Supplementary file 1 [file ijerph-16-03859-s001.zip › Supplementary files/Figure S3 - Forest plot different type.tif]

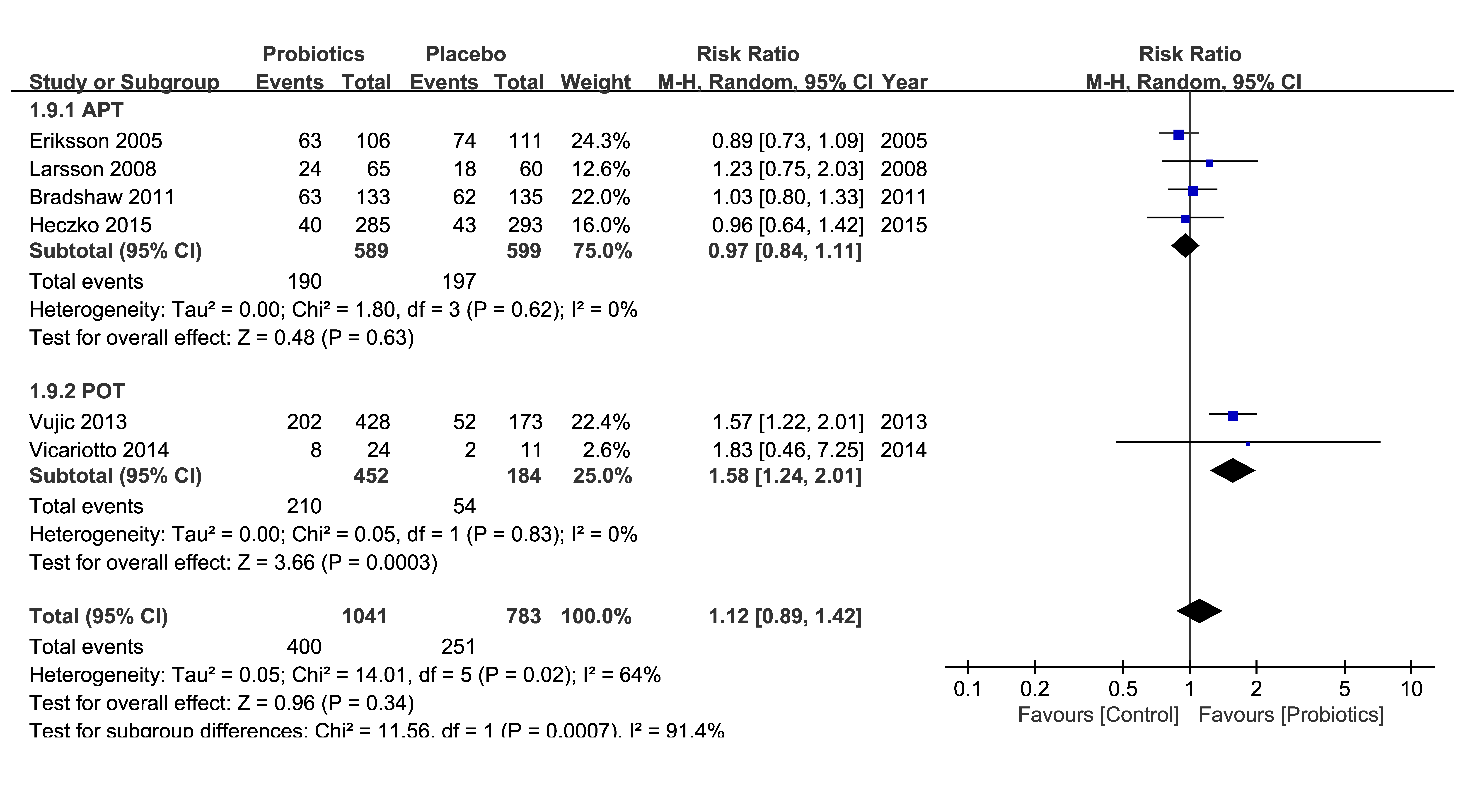

Supplement: Supplementary file 1 [file ijerph-16-03859-s001.zip › Supplementary files/Figure S4 - long-term.tif]

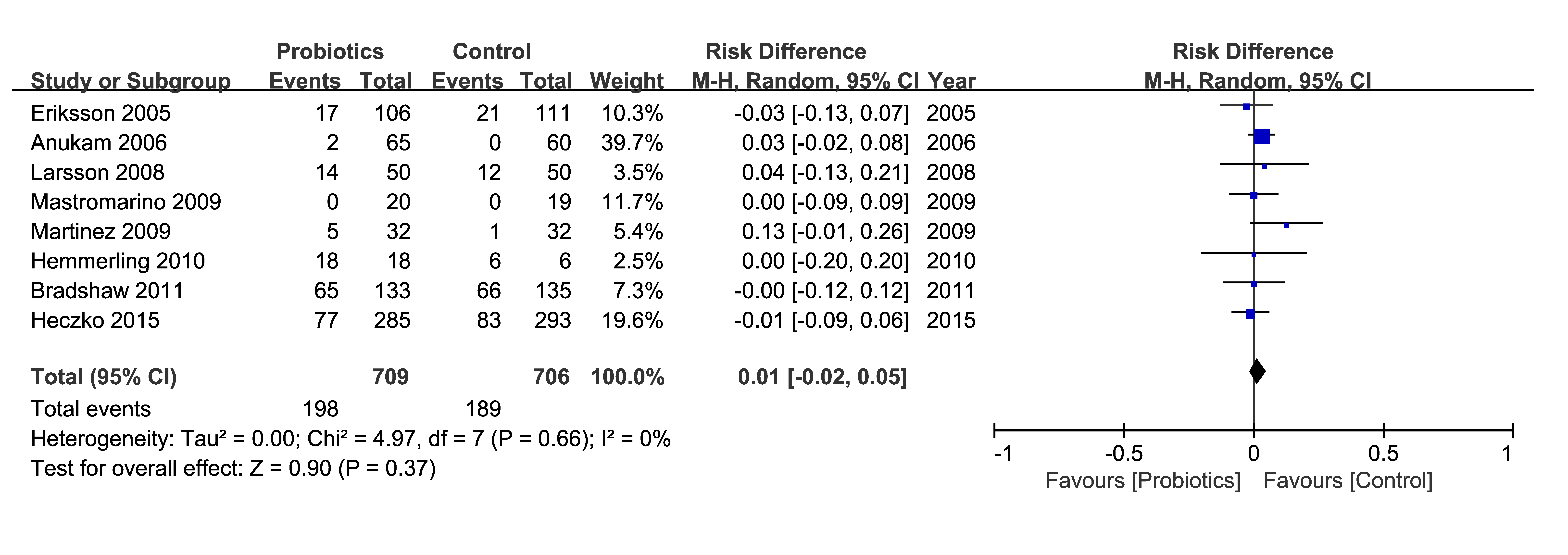

Supplement: Supplementary file 1 [file ijerph-16-03859-s001.zip › Supplementary files/Figure S5 - safety.tif]

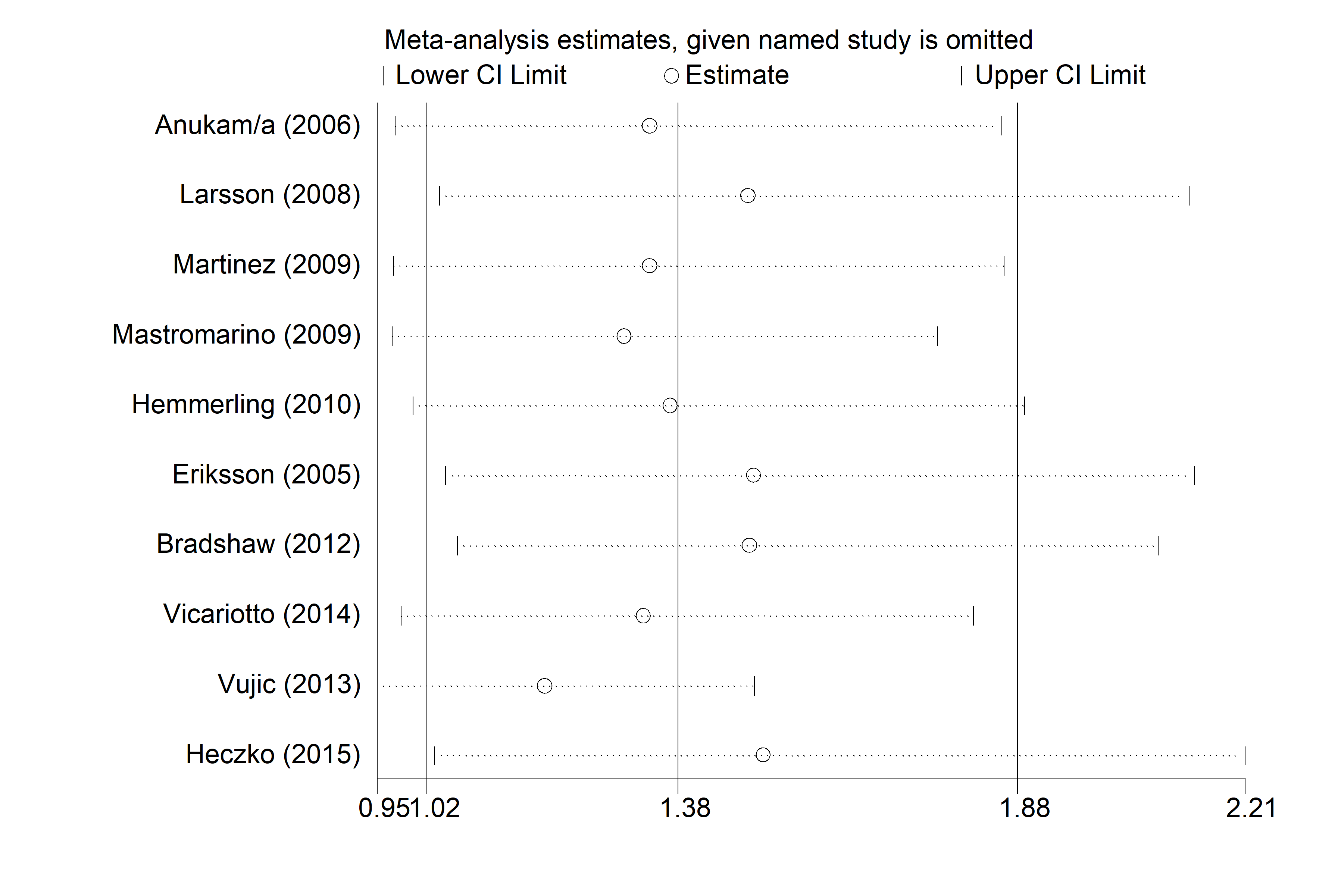

Supplement: Supplementary file 1 [file ijerph-16-03859-s001.zip › Supplementary files/Figure S7.tif]

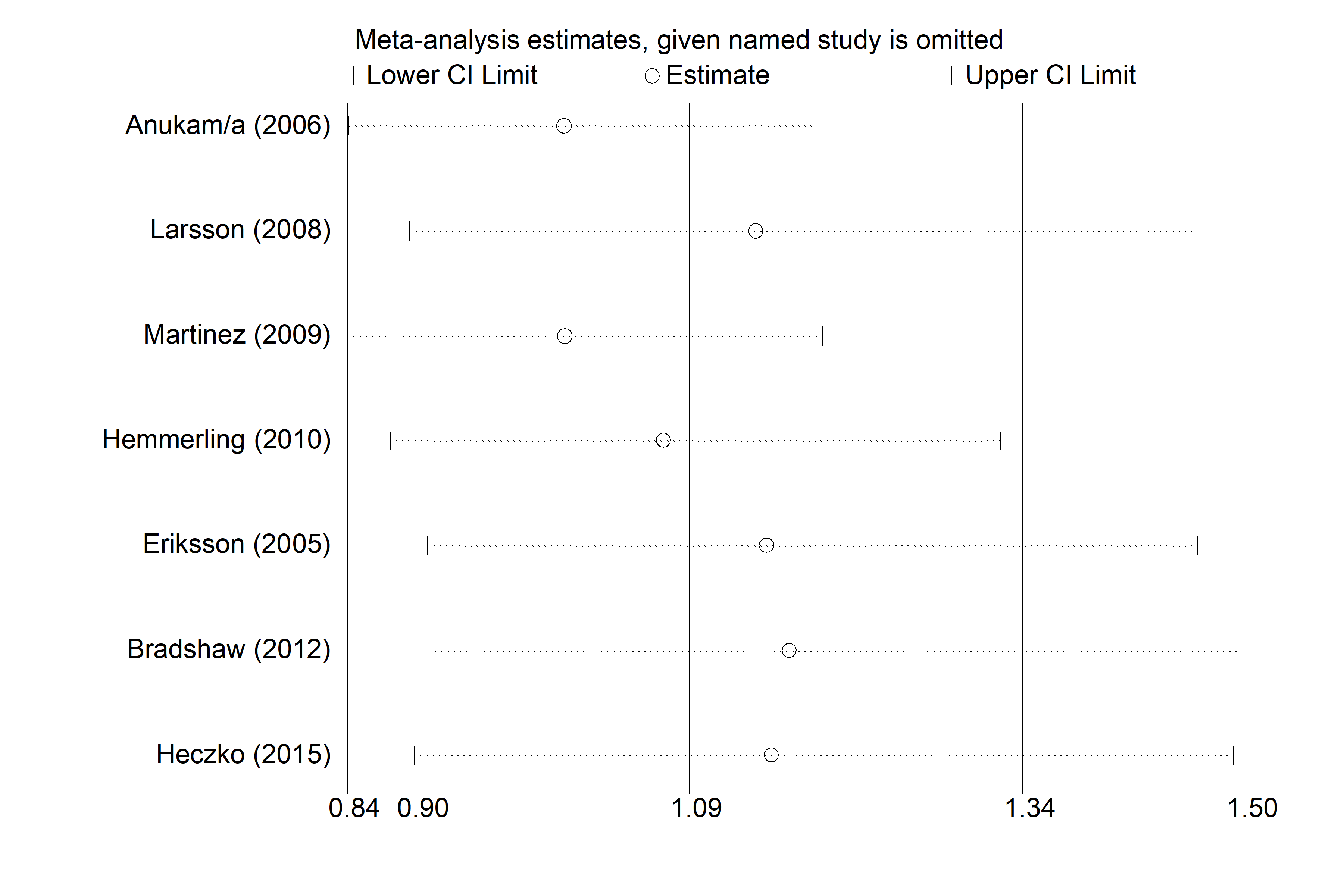

Supplement: Supplementary file 1 [file ijerph-16-03859-s001.zip › Supplementary files/Figure S8-APT.tif]

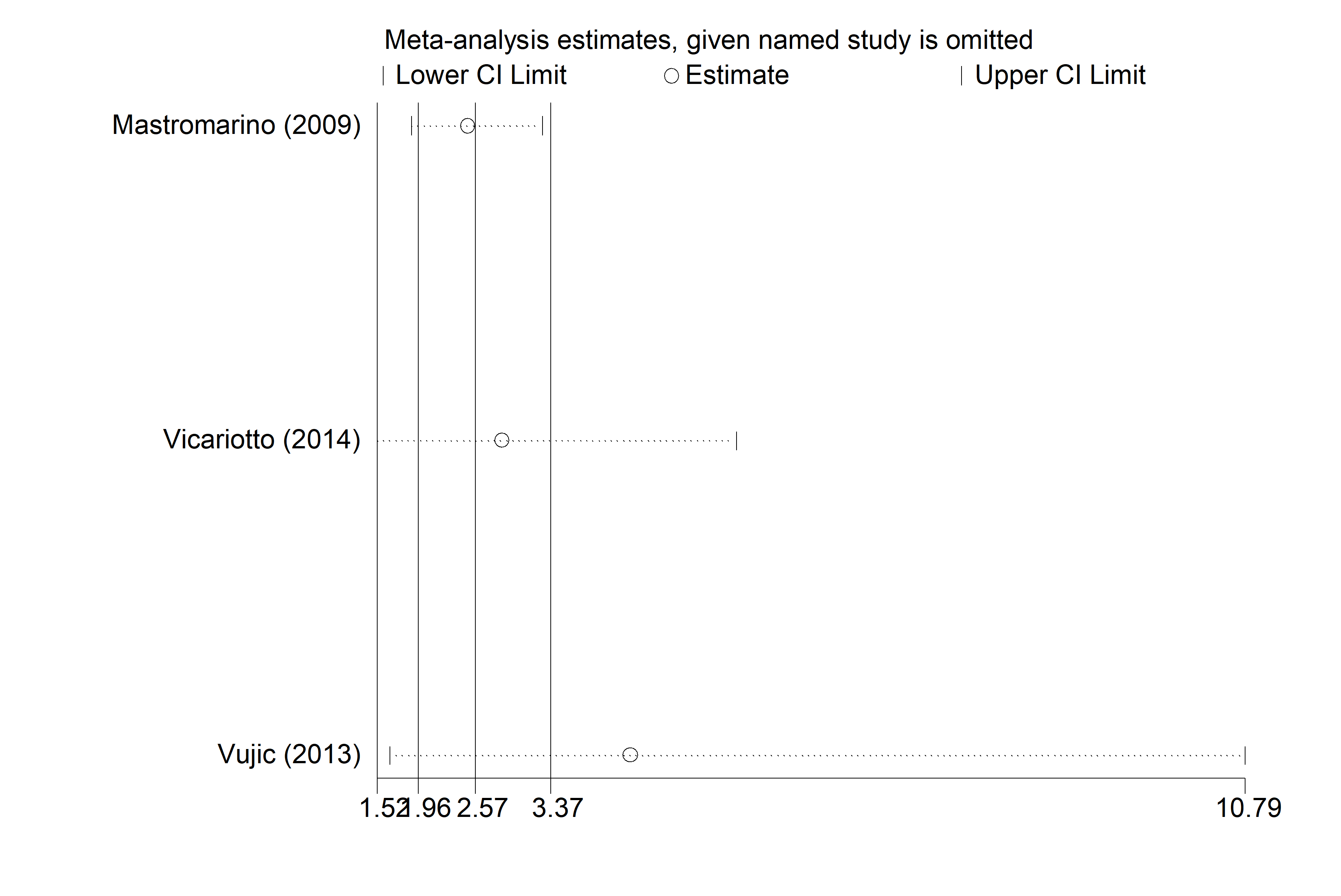

Supplement: Supplementary file 1 [file ijerph-16-03859-s001.zip › Supplementary files/Figure S9-POT.tif]
